# Up-To-Date Analysis of the Extraction Methods for Anthocyanins: Principles of the Techniques, Optimization, Technical Progress, and Industrial Application

**DOI:** 10.3390/antiox11020286

**Published:** 2022-01-30

**Authors:** Noelia Tena, Agustin G. Asuero

**Affiliations:** Departamento de Química Analítica, Facultad de Farmacia, Universidad de Sevilla, Prof. García González 2, 41012 Sevilla, Spain; asuero@us.es

**Keywords:** anthocyanins, non-conventional extraction techniques, anthocyanin yields, industrial application, optimization

## Abstract

Nowadays, food industries are concerned about satisfying legal requirements related to waste policy and environmental protection. In addition, they take steps to ensure food safety and quality products that have high nutritional properties. Anthocyanins are considered high added-value compounds due to their sensory qualities, colors, and nutritional properties; they are considered bioactive ingredients. They are found in high concentrations in many by-products across the food industry. Thus, the non-conventional extraction techniques presented here are useful in satisfying the current food industry requirements. However, selecting more convenient extraction techniques is not easy. Multiple factors are implicated in the decision. In this review, we compile the most recent applications (since 2015) used to extract anthocyanins from different natural matrices, via conventional and non-conventional extraction techniques. We analyze the main advantages and disadvantages of anthocyanin extraction techniques from different natural matrices and discuss the selection criteria for sustainability of the processes. We present an up-to-date analysis of the principles of the techniques and an optimization of the extraction conditions, technical progress, and industrial applications. Finally, we provide a critical comparison between these techniques and some recommendations, to select and optimize the techniques for industrial applications.

## 1. Introduction

**Figure 1:**
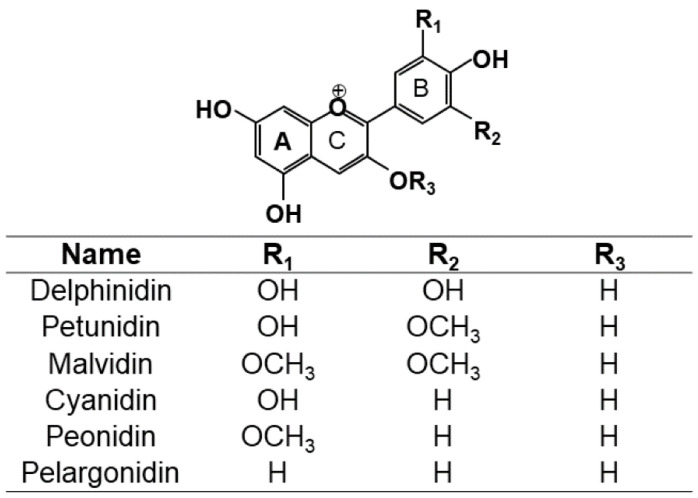
Structure of anthocyanins R_3_ = sugar and anthocyanidins R_3_ = H (taken from Tena et al. [2]).

In recent years, interest in food quality has increased. Consumers are increasingly becoming aware about the correlation between nutrition and health. Thus, many consumers are opting for foods that contain bioactive ingredients, which have health-promoting properties [1]. Vegetables and fruits have high amounts of antioxidant compounds that are considered bioactive ingredients. Different chemical compounds present in vegetables, fruits, and flowers have antioxidant properties. Some of these compounds belong to the group of anthocyanins, which are not only antioxidant compounds, but are also natural colorants (with aglycones of anthocyanins being the most common plant pigments). The properties of anthocyanins have been extensively studied in the literature [2,3,4,5,6,7,8,9,10]. These properties are directly related to their chemical structures. A flavylium cation acts as an acid, and it gives anthocyanins a high chemical reactivity. The structures and properties of anthocyanins depend on different factors, such as temperature, pH, and solvent. Figure 1 shows the base structure of anthocyanins. These factors must be considered in the extraction processes to minimize changes in the quality and activity of the resulting extract [2,3].

The interest in anthocyanins can be attributed to the fact that they can be used as natural coloring agents rather than as synthetic coloring agents [11,12,13,14]. Anthocyanins have attractive colors, ranging from red to purple, and their use as food coloring agents has been authorized under code E163 by the European Food Safety Authority (EFSA) [11,15]. This has expanded the use of these natural coloring agents at the industrial level; they can be included in the food industry because they are innocuous and safe molecules. Moreover, anthocyanins have gained attention due to their potential health benefits, e.g., (i) anti-inflammatory, antioxidant, anti-diabetic, and anti-cancer properties; (ii) preventing cardiovascular diseases, neurological disorders, obesity, and for eye health; (iii) improving the gut microbiome; and (iv) decreasing H_2_O_2_-induced cell apoptosis of the human normal liver cell (LO2 cell) line [2,16,17,18]. Thus, anthocyanins provide excellent added value to foods due to their dual nature as coloring agents and antioxidants, with beneficial effects on health. Therefore, they are determining factors in the quality and value of fruits and vegetables, and in processed food derived from those. In addition, they are widely used in the food and cosmetics industries.

However, their supply currently depends, to a large extent, on the complex extractions of these compounds from the matrix. At present, expensive raw materials and production technologies make the extraction of natural anthocyanins relatively expensive [1,19]. For this reason, new sources of anthocyanins are being studied, in order to extract these compounds at lower costs. Recent studies have explored the extraction performances of anthocyanins in different matrices, such as microalgae [20], potatoes [21,22], and rice [19,23]. In order to comply with current regulations regarding environmental sustainability, to improve economic performance and reduce waste in the food industry, there is growing interest in investigating the possibility of extracting anthocyanins from by-products and waste generated at different industrial food productions [17,24,25,26,27,28,29]. Anthocyanins recovered from food waste could have high potential in being used in different food and biotechnological applications, e.g., as food supplements, nutraceuticals, and/or food additives.

The food industry is currently looking for new sources of bioactive compounds, such as anthocyanins, to meet consumer demands. By-products and waste from some food industries represent low-cost sources of anthocyanins that are of interest to the industry. The extraction methods applied to anthocyanin extraction in different natural matrices have been extensively studied [6,17,30,31,32,33,34,35]. Conventional techniques, such as maceration and heat-assisted extraction (HAE), do not require sophisticated instrumentation and are easy to apply at the industrial level. However, they have a number of limitations, such as the following: the toxicity of the solvents used, possible solvent residues in the extracts, safety risks associated with the use of large volumes of solvents, deterioration of the extracts due to heating, and low yields in the extraction of anthocyanins. The latter limitation may be due to the fact that anthocyanins are found in the vacuoles of plant cells. In order to extract them with cost-effective yields, it is necessary to apply extraction methods that reduce the mass transfer resistance of the plant cell wall. To avoid the resistance of the cell wall, emerging extraction technologies have been proposed, such as ultrasound-assisted extraction (UAE), microwave-assisted extraction (MAE), supercritical fluid extraction (SFE), high-pressure liquid extraction (HPLE), pulsed electric fields (PEFE), high voltage electrical discharge (HVED), and enzyme assisted extraction (EAE). These techniques require more sophisticated instrumentation than conventional techniques, but have some advantages: in general, they do not use heat for extraction, reducing energy costs, or improving the stability of the extracts. In addition, the volume of solvent used is lower (or zero) compared to conventional techniques, reducing greenhouse gas emissions and complying with the legal requirements of green chemistry. Thus, selecting the most appropriated extraction techniques is the primary goal for many industries, so that they can increase profitability by decreasing energy costs, and be responsible with the “green chemistry”.

This review provides a critical comparison on the extraction methods used for anthocyanin extraction in recent years (2015 onwards). We classified the extraction methods into conventional and non-conventional techniques. For each one, we describe the principles of the techniques, the optimal parameters for anthocyanin extraction (with concrete examples), the most recent innovations in the techniques, and we comment on the industrial applications.

## 2. Extraction Methods Used to Extract Anthocyanins

The first step is extraction. In this way, the desired natural products can be separated from the raw materials. Solvent extraction, distillation method, pressing, and sublimation are some extraction methods, according to the extraction principles. The selection of the extraction process, to extract anthocyanins, is based on the preservation of the stability and shelf life of these compounds, which is directly related to the beneficial properties that these compounds provide. Sometimes, prior to applying the selected extraction technique, it is necessary to eliminate other compounds present in the sample matrix that may hinder the extraction of the anthocyanins, such as lipids, proteins, or contaminants. In this section, the conventional extraction techniques applied to extract anthocyanins are described and classified, with their advantages and disadvantages highlighted.

### 2.1. Conventional Extraction Techniques

Depending on the procedure used to mix the powdered solid sample with the solvent, the conventional techniques are commonly classify as: (i) maceration, when the powdered crude sample is mixed with solvent; (ii) infusion, when a maceration is carried out with water; (iii) digestion, when a maceration is carried out with mild heating, also known as heat-assisted extraction (HAE); (iv) decoction, when an infusion is made with boiling water; (v) percolation and filtration, when the powdered sample is mixed with a continuously renewed solvent in a percolator, with a filtration process applied afterwards. More recently, the Soxhlet extraction technique emerged, which consists of mixing the powdered solid with the solvent inside a “Soxhlet apparatus”, allowing continuous cyclical repetitions of the extractions during a controlled period of time.

These techniques are based on the use of different types of solvents and/or heat. Considering the law that “like dissolves like”, the solvents commonly used to extract anthocyanins are: methanol, ethanol, water, acetone, or mixtures thereof. Acid solutions are often added to these solvents to help stabilize the flavylium cation, which is stable in highly acidic conditions (pH ~ 3). To achieve this, the use of weak acids (e.g., formic acid, citric acid, or acetic acid) is recommended, since the use of strong concentrated acids may lead to destabilizing the anthocyanin molecule. In view of the polar structure of the anthocyanins, the addition of water to the solvent mixture can improve the extractive yield.

In addition to the solvents, the powder size of the solid, the solvent-to-solid ratio, and the time and temperature of the extraction are other analytical parameters that should be optimized to ensure the maximum yields. Different authors have optimized some of these analytical parameters to extract the maximum yield of total anthocyanin content (TAC) in different natural matrices. Paludo et al. [36] concluded that the optimal conditions for extracting anthocyanins and phenolic compounds from the skin and seed of Jabuticaba (*Plinia cauliflora*) fruits, respectively, was a solvent mixture of methanol/water/acetic acid (80:20:0.5 *v*/*v*/*v*), with a solid–liquid ratio of 0.01 g/mL, with two hours of constant agitation. Other authors consider the use of temperature necessary to optimize the extraction yield, as is the case of Albuquerque et al. [11], who claim that the optimal conditions to extract anthocyanins in the skins of Jabuticaba fruits involved a solvent mixture of ethanol (9.1% *v*/*v*) acidified with citric acid at pH 3, mixed and centrifuged with the powdered sample (~20 mesh), in a solid–liquid ratio of 50 g/L during 21.8 min at 47.1 °C. They conclude that the total amount of anthocyanins extracted increase with mild temperatures and decrease with high ethanol concentrations and with long times of extraction. The latter could be explained by the fact that long times could lead to the breakdown of the structures of sensitive compounds, such as cyanidin-3-O-glucoside [11]. Other studies carried out with other natural matrices found similar extraction conditions in the optimization of anthocyanin extraction. Thus, Demirdöven et al. [37] determined that a solvent mixture of ethanol (42.39% *v*/*v*) acidified with formic acid in a solid–liquid ratio of (1:3 *w*/*v*) heated at 40 °C for 75 min were optimal conditions to extract anthocyanins in red cabbage. Backes et. [38] affirmed that the optimal analytical parameters to improve the yield of TAC in fig skin was a solvent mixture of ethanol (100% *v*/*v*) acidified with citric acid (pH = 3), mixed and centrifuged with the powdered sample (~20 mesh) in a solid–liquid ratio of 50 g/L for 13.74 min at 35.64 °C. These authors revealed that the high content of ethanol and the temperature increase the yield of the extraction. In the particular case of cyaniding-3-rutinoside, the maximum extraction was obtained when 100% of ethanol was used, in contrast to what was stated by Albuquerque et al. [11]. It is well-known that high values of ethanol in the solvents increase the extraction of bioactive compounds from plant materials. However, these studies pointed out the importance of the amount of ethanol in the solvent mixture in the selective extractions of individual anthocyanins [11,12,37]

These studies show that, at the laboratory level, the analytical parameter, solid–liquid S/L ratio, has little effect on TCA extraction [12]. However, Backes et al. [38] conducted a study to optimize the solid-to-liquid (S/L) ratio in order to apply HAE at the industrial scale. They determined that a S/L ratio higher than 200 g/L does not allow a homogenous mixture. Furthermore, they established that, depending on the TAC in the natural material, the optimal S/L ratio changes, since highly concentrated samples will saturate the solvent earlier and need lower S/L than less concentrated samples. Their results applied to fig skin concluded that a S/L ratio higher that 100 g/L provoked saturation of the solvent, leading to a decrease in cyanidin-3-rutinoside levels. Fernandes et al. [12] affirmed that, in basil leaves, when the S/L ratio increased from 15 to 30 g/L, any significant proportional decrease was not observed. Thus, they concluded that ratios lower than 30 g/L do not provoke saturation in the extraction of anthocyanins from basil leaves.

These types of extractions are currently the most widely used in the industry, particularly in natural dye industries, likely because these extraction methods have low instrumentation costs. Despite their wide use, these methods also have some disadvantage: (i) high energy consumption; (ii) the use of environmentally unfriendly organic solvents; (iii) the need for expensive and high purity solvents; (iv) the use of a large volume of solvents; (v) the application of a long extraction time to extract compounds with lower yields; (vi) the need for moderate–high temperatures in some cases, which could cause deterioration of antioxidants; (vii) the need for evaporation of a huge amount of solvents; and (viii) the low selectivity of extraction [24,29,39]. Thus, conventional extraction techniques are currently applied in the industry. However, they do not provide high yields in the extraction of anthocyanins, although the yield can improve by increasing the temperature of the extraction. It can also provoke change in the color or in the properties of the extract. The most important factors to optimize are the S/L ratios, which depend on the TAC in the matrix and the solvent used for the extraction. The optimization of the solvent amount is essential to minimize the use of contaminants and work, in agreement with the legal requirements, and in order to reduce the evaporation costs.

### 2.2. Non-Conventional Extraction Techniques

To overcome the above-mentioned disadvantages of conventional extraction methods, new and promising extraction techniques have been introduced over the year. These techniques are more environmentally-friendly and have important industrial focuses, as they aim to improve the extraction efficiency and yield. However, they have not been employed on a massive scale yet. Among these extraction methods, the most applied techniques to extract anthocyanins are: ultrasound-assisted extraction (UAE), microwave-assisted extraction (MAE), supercritical fluid extraction (SFE), high-pressure liquid extraction (HPLE), pulsed electric fields (PEFE), high voltage electrical discharge (HVED), and enzyme assisted extraction (EAE):

#### 2.2.1. Ultrasound-Assisted Extraction (UAE)

**Figure 2:**
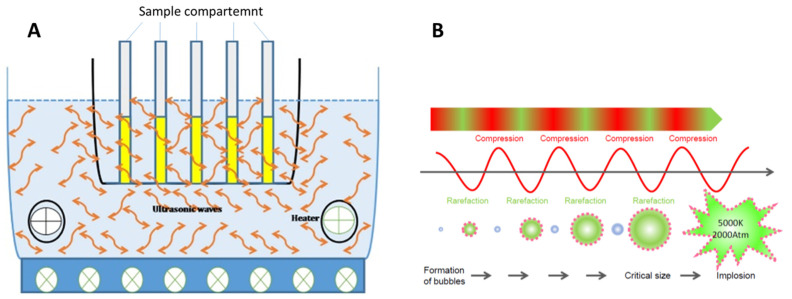
(**A**) Schematic representation of ultrasound-assisted extraction (UAE) equipment, in which a fixed amount of crushed natural material is placed inside the sample compartment. (**B**) Explanation of the formation of the bubbles by alternative compression–rarefaction effects generated by ultrasound waves. (**A**) adapted from Belwal et al. [29], with permission from Elsevier, and (**B**) taken from Morata et al. [10]).

The UAE technique is based on an ultrasound force that is able to break the cell wall due to that cavitation phenomenon that occurs in the tissue of the sample [10,40]. Figure 2 shows a schematic representation of ultrasound-assisted extraction (UAE) equipment, with an explanation about the cavitation process that occurs as a consequence of the ultrasound force. The ultrasound moves through the liquid as waves formed by a process of compression and rarefaction, generating cavitation bubbles with the liquid. The sizes of the bubbles increase with the repetition of a few cycles of compression and rarefaction, up to a critical size (Figure 2B). Then, the bursting of the bubbles, producing a large amount of energy in the form of pressure and temperature, destroys the cell walls, improving the mass transport of the anthocyanins from the plant cell walls to the solvent [41]. UAE could be performed using an ultrasonic bath (BUE) or ultrasonic probe (PUE). Bath ultrasonic extraction (BUE) is more economical and easier to handle. However, the energy produced is not homogeneously distributed in the bath, reducing the efficiency of the extraction. In addition, it has lower reproducibility than PUE. Probe ultrasonic extraction (PUE) consists of a probe connected to a transducer. The probe is immersed in the extraction vessel and disperses the ultrasound in the media with a minimum energy loss. PUE provides higher ultrasonic intensity than the bath system. In PUE, the ultrasound energy is concentrated in a specific zone of the sample, making the extraction more efficient. PUE is commonly preferred to extract bioactive compounds when compared with the bath system [17]. Sabino et al. [42] carried out a comparative study between BUE, PUE, and pressurized liquid extraction (PLE) to determine which technique was most efficient at extracting anthocyanins from jambolan fruit. They concluded that PUE promoted the largest recovery of anthocyanins compared to PLE and BUE. In addition, they pointed out that the three studied techniques extracted the same type of anthocyanin [42].

UAE is widely used in the extraction of bioactive compounds in different natural matrices [10,21,29,41,42,43,44,45]. However, to obtain the maximum yield of anthocyanins by UAE, it is necessary to optimize the extraction conditions. The parameters to be optimized are temperature, time, solvent composition, the liquid/solid (L/S) ratio, moisture content in the sample, particle size, and ultrasound power [21]. Table 1 shows some current examples selected from the literature. It shows the optimal extraction conditions in different natural matrices and the yields obtained by UAE. Demirdöven et al. [37] optimized the extraction conditions, time, temperature and solvent composition (percentage of ethanol) to maximize the efficiency of anthocyanin extraction from red cabbage by UAE (Table 1). 

Espada-Bellido et al. [45] showed that temperature and solvent concentrations were the most influential extraction conditions for the extraction of anthocyanins from black mulberry pulps by UAE. However, some studies have demonstrated that the ultrasonic power or force and the pulse cycle applied to the extraction play important roles in the yields of the anthocyanins extractions. Thus, Ravanfar et al. [43], in a study involving red cabbage, reported that time, temperature, and the ultrasonic power were the parameters that most affected the extraction yield, while the pulsation during sonication had no significant effect [43]. Agcam et al. [44] demonstrated the importance of the ultrasonic power in the increment of the anthocyanin extracted from black carrot pomace. Their study also demonstrated the synergy of the combination of ultrasonication and temperature to increase the yield of anthocyanin extraction in black carrot pomace [44]. Different studies have revealed that the ultrasound power, defined by its frequency and intensity, together with time and temperature, are parameters that have a direct effect on the efficiency and yield of anthocyanins extraction [11,41].

Studies have demonstrated that UAE increases the yield of the extraction of antioxidant compounds versus conventional techniques (by at least 20%) and increases the antioxidant activities of the extracts, improving the extraction efficiency of antioxidant compounds [41]. Thus, Backes et al. [37] and Demirdöven et al. [38] showed that they obtained better yields by UAE than by HAE when extracting anthocyanins from red cabbage and fig peel, respectively (Table 1). However, the study carried out by Albuquerque et al. [11] revealed that HAE gave better yields (76 mg/g) than UAE (32 mg/g), when anthocyanins were extracted from the epicarp of Jabuticaba. They attribute these results to the less agitation (and, therefore, less homogenization) between the solvent and the sample in the UAE extraction than in the HAE. Other authors have obtained similar results. These results indicate that the nature of the anthocyanin (e.g., its anthocyanidin and sugar molecule) may also be a factor influencing UAE extraction [11]. 

Backes et al. [38] not only obtained the highest yield for fig peel extraction by EAU than by HAE and MAE, but they also showed that the purity of fig peel extract increased as the L/S ratio decreased [38]. Mane et al. [49], as well as other authors, confirmed that, in the extraction of anthocyanins from the purple potato by UAE, the extraction conditions interfering with the extraction yield were the potato shape, time, solvent composition, solvent ratio, and ultrasound power. They affirmed that a higher organic solvent:water ratio increases the TAC extracted. 

UAE is proven to be a useful technology for the extraction of anthocyanins. However, overly-long extractions can destroy these bioactive compounds. More recently, some modifications to the ultrasound-assisted technique have been introduced to increase extraction performance and to reduce time and energy consumption. Mane et al. [49] explored the effect of the combination of UAE with a previous microwave treatment of the sample—in this case, potato. They observed that this combination decreased the volume of the solvent and the time needed to obtain the same extraction yield [49]. Another proposal is the case of the pulsed ultrasound assisted technique (PUAE), which, instead of applying the ultrasound continuously, it is applied intermittently, producing less heat and saving costs [50]. Another alternative has been the combination of Ultra-Turrax with ultrasound-assisted extraction—Ultra-Turrax-based ultrasound-assisted extraction (UT-UAE). Ultra-Turrax allows producing a narrow and uniform particle size distribution that can increase the extraction speed [51]. Another innovation is the combination of an ionic liquid with UAE—ionic liquid-based ultrasound-assisted extraction (IL-UAE). This combination produces a synergy effect that increases the extraction efficiency due to the characteristic of the ionic liquid, such as high polarity, high ionic conductivity, and chemical stability [52].

From the results shown in this section, it can be concluded that UAE, under optimal conditions, can be considered an easy and economical tool for the extraction of anthocyanins, considering that the anthocyanins are labile at a basic pH, at high temperatures, and when exposed to light [2]. The extraction of anthocyanins should be carried out by avoiding the exposition of the sample to these factors; thus, faster extraction techniques are recommended when compared with conventional techniques, in order to avoid deterioration of the anthocyanins. The UAE, using a cavitation process, allows the solvent to easily penetrate into the solid matrix by increasing the mass transfer between the solid matrix and the solvent. This allows higher yields to be obtained in short periods of time and at low temperatures. Consequently, this technique is not only ideal because it is faster, but it also consumes less solvent, is more environmentally friendly, and has a lower cost with higher yields compared to conventional extraction techniques. In addition, this technique is simple (Figure 2) and does not require complex maintenance [10,21]. At the industrial scale, UAS is a promising technique to displace conventional techniques, due to less extraction times, higher extraction yields, and lower operating temperatures.

#### 2.2.2. Microwave-Assisted Extraction (MAE)

**Figure 3:**
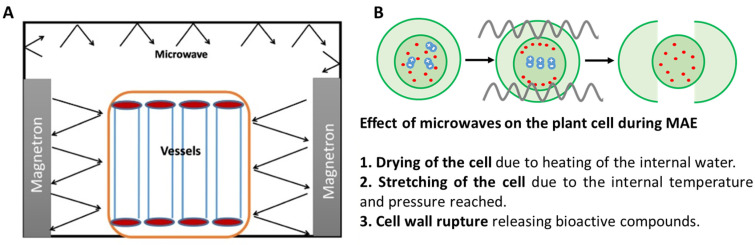
(**A**) Schematic representation of microwave-assisted extraction (MAE) equipment, in which a fixed amount of crushed natural matrix is placed inside the vessels, together with the solvent. (**B**) Explanation of how microwave heating of the cell by ionic conduction and dipolar rotation causes cell wall rupture. ((**A**) adapted from Belwal et al. [29], with permission from Elsevier).

The MAE technique uses electromagnetic radiation energy in the microwave region. This energy is absorbed by the polar molecules present in the solvent and the food, producing a dipolar rotation in the molecules and the migration of ions. Microwaves act selectively on plant cells, vaporizing the water in the matrix and, thereby, generating high pressure in the cell wall. This effect produces heat, causes changes in the physical properties of the cell wall, and eventually leads to cell wall rupture [29,41,53]. Thus, the solvent penetrates more easily into the plant cell, favoring the mass transport from the cell to the solvent. The heat produced during this process is transferred from the inside of the plant cell to the outside, which occurs in the opposite direction in UAE. Figure 3 shows a schematic representation of microwave-assisted extraction (MAE) equipment, along with an explanation about the heating produced by ionic conduction and dipole rotation.

The extraction conditions that should be optimized to increase the yield of the bioactive compounds by MAE are: the composition of solvent, which must be sufficiently polar to absorb the microwaves radiation, L/S ratio, temperature, time, microwaves power, and particle size of the matrix. MAE has been used to extract anthocyanins in recent studies. Table 2 shows some recent examples selected from the literature on the application of MAE, to extract anthocyanins from different natural matrices.

Xue et al. [54] applied MAE to extract anthocyanins from blueberries. They studied the effects of microwave powers on the yield of extraction and observed that rising microwave powers had little effect on the distribution of microwave energy, but increased the temperature in the center location in the vessels. They concluded that the highest anthocyanin yield was obtained at a critical temperature of 50.75 °C and suggested that the control of microwave power contributes to the improvement of anthocyanin yield and efficiency of microwave energy [54]. Farzaneh and Carvalho [55] applied MAE to extract anthocyanin from *Lavandula pedunculata* L. fresh plants. They studied the effect of microwave irradiation power, irradiation time, and L/S ratio on the yield. They observed that the highest antioxidant activity was obtained with a lower power (300 W) and time of irradiation (107.3 s) and a higher L/S ratio (34.807 mL/g) than those applied to obtain the highest TAC (Table 2) [55]. These results could be explained by the fact that a higher irradiation power and time increases the yield of the extraction, as well as increases the temperature of the sample. It could also damage the thermally labile antioxidants, which might result in the reduction of their antioxidant activities. Thus, Sun et al. [56] applied MAE to extract anthocyanins from powdered blueberry. They demonstrated that the acquirement and degradation of anthocyanins occur simultaneously with exponential trends. They determined that the lowest degradation of anthocyanins were products at a temperature of 53.3 °C. Lower temperatures favored the self-aggregation of the anthocyanins and higher temperatures provoked the degradation of them. They proved that the acquired anthocyanins were higher and the degradation was lower by MAE than by hot reflux extraction (HRE), a conventional technique [56]. Thus, microwave power and the time of irradiation are complementary variables that influence the extraction process. High power can increase the heating effect decreasing the microwave irradiation time and increasing the yield, but it also might provoke a degradation of thermally labile compounds [41]. Thus, Xue et al. [57] optimized the irradiation time and the resulting temperature to achieve the highest yield of anthocyanins from cranberry. They concluded that an irradiation time of 8 s and an extraction temperature of 50 °C provided the highest yield (Table 1). Their results showed that increasing the temperature was beneficial for anthocyanin extraction when the extraction temperature was below 323 K (50 °C) [57]. Despite the need to control and optimize the thermal conditions under which MAE is carried out to avoid a negative effect on the antioxidant properties and the color of the extract obtained, several studies have been carried out to explore the possible application of this technique in the extraction of anthocyanins [Table 2].

Several studies have shown that MAE has some advantages compared to conventional extraction [58,59,60,61,62]. These studies claim that MAE lasts for less time and consumes less solvent [58], and allows higher extraction yields than a conventional extraction [41,56]. In addition, it is an energy-saving method due to its short processing time. However, the main limitation of this technology is the negative effect of heating, caused by microwave energy, on the properties of anthocyanins.

Nowadays, in order to overcome the possible limitations of the MAE, some modifications have been introduced, such as working at low pressures or working at atmospheric pressure. Changes have also been made to the type of solvent used in MAE. Applications have been developed in which oxygen flow or different aqueous phases are used for extraction. Some of the most current proposals could be classified as the following [30]:

**MAE for thermolabile substances,** which include: nitrogen-protected microwave-assisted extraction (NPMAE) and vacuum microwave-assisted extraction (VMAE). This is the most convenient alternative to extract anthocyanins. These techniques are based on using low oxygen content inside the extraction tanks, combined with moderate or low temperatures. In this way, they can avoid degradation of thermolabile, and oxygen-sensitive plant phytochemicals compared to conventional MAE [30,41].

**Microwave heating extraction,** which includes: focused microwave-assisted Soxhlet extraction (FMASE), ultrasonic microwave-assisted extraction (UMAE), microwave hydro-distillation (MWHD or MAHD), and microwave steam distillation (MSD).

**Green extraction without solvent,** which includes: solvent-free microwave extraction (SFME), vacuum microwave hydro-distillation (VMHD), microwave hydro-diffusion and gravity (MHG).

**Microwave extraction with no solvent pressure,** which includes: pressurized solvent-free microwave extraction (PSFME).

Odabas and Koca [63] proposed microwave assisted aqueous two-phase extraction (MA-ATPE) to extract anthocyanins from *Rosa pimpinellifolia* L. fruits. They applied MA-ATPE to simultaneously extract and purify the anthocyanins with an ethanol/ammonium sulfate aqueous two-phase system. The optimal anthocyanin yields (1373.04 mg C3G/g DW) were achieved in conditions of 0% HCl (w:w), 26.85% ethanol (*w*/*w*),19.15% ammonium sulfate (*w*/*w*), L/S ratio 40, 17.29 min, 60 °C and 400 W. They showed that the purity of the anthocyanin extract was 1.65 times higher with MA-ATPE than with MAE, using 80% ethanol [63]. SFME is also an applied alternative to extract flavonoids. This alternative is a green technique that uses the in-situ water in plant cells to absorb the microwave energy and break the cell wall. The extraction could be carried out at atmospheric pressure. Some applications of this technique can be found in the literature for the extraction of flavonoids from different matrices, such as onion [64] or mandarin leaves [65]. Another possibility consists of applied SFME under pressure PSFME. Thus, Michel et al. [66] applied this technique to extract antioxidants from sea buckthorn (*Hippophae rhamnoides* L.) berries. They demonstrated that PSFME, compared to other conventional extraction techniques, such as pressing, maceration, and pressurized liquid extractions lead to the most active and richest extract in phenolic content. Molecules, such as quercetin and isorhamnetin, were extracted in this way, in contrast to other previously applied techniques [66]. Solvent-free hydrodiffusion and gravity MHG have also been applied to extract antioxidants from food by-products from sea buckthorn obtaining similar results [25].

This technique and its variants work well in terms of solvent consumption, extraction time, and extraction yield, and they are considered as possible substitutes for conventional methods. The yield of natural components from vegetable matrices, for implementation as food ingredients, must be carried out under the best extraction conditions, to promote application at an industrial scale, competing against the low economic costs of producing artificial compounds. The characteristics of the MAE and its effectiveness cannot be applied in a generalized way to all matrices, demanding specific optimization for each particular case. In addition, some factors might affect anthocyanin stability and, so, their deterioration rate, reinforcing the importance of determining the conditions that maximize the extraction yield of these compounds. Thus, this technique could be well applied at industrial scale and some studies demonstrate that MAE has been applied to extract anthocyanins from food waste, such as by-products of the wine industry and grape juice [28,29]. However, there are few studies on the application of this technique at an industrial scale. This could be explained by critical points in industrial microwave design. Therefore, to promote the use of MAE in the food and drug industries, it is necessary that research points to the technical issues related to the design of microwave extractors and their suitability for the isolation of bioactive components from the vegetal matrix [30].

#### 2.2.3. Pressure Fluids Extraction Techniques

Extraction under pressure is another alternative used among the non-conventional methods to facilitate mass transfer from the inside to the outside of the plant cell, in addition to the use of electromagnetic radiation in the ultrasound or microwave region. In the case of high-pressure fluid extractions, a reduction in extraction times and in the amount of solvent used has been observed. In addition, the pressure applied influences the selectivity of the extraction. Currently, different techniques based on pressurized fluid extractions have been applied for anthocyanin extraction. They can be classified as follows: supercritical fluid extraction (SFE), pressurized liquid extraction (PLE), and high pressure liquid extraction (HPLE).

##### Supercritical Fluid Extraction (SFE)

**Figure 4:**
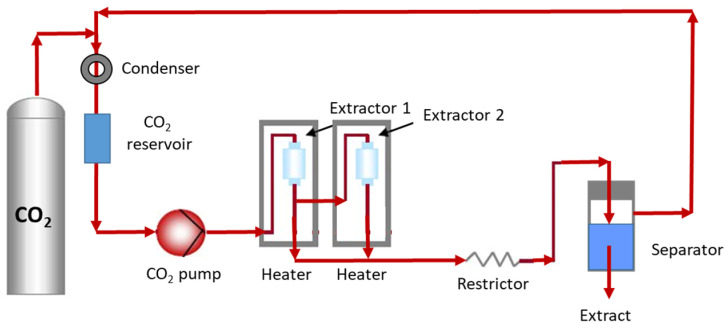
Schematic representation of a supercritical fluid extraction (SFE) equipment, in which a fixed amount of crushed sample is placed inside the extractor 1 and 2.

SFE has been widely applied in the last decades, as it is considered one of the most sustainable green technologies. This technique commonly uses supercritical CO_2_ (critical temperature (CT) = 31.3 °C; critical pressure (CP) = 72.9 atm) as solvent, since it is nontoxic, not very expensive, preserves the extracts from atmospheric oxidation, and has a moderate CT. The disadvantage of this solvent is that it is non-polar. This is why a co-solvent can be added to promote the extraction of polar compounds, as in the case of anthocyanins [31]. The most common co-solvents used are ethanol, methanol, and aqueous solutions of these alcohols, in concentration within 1% to 15% [67]. In general, SFE and PLE are carried out under medium-to-high pressures. However, SFE operates using solvents at temperatures and pressures above their critical points, and PLE is based on the use of liquids at temperatures above their normal boiling points. SFE uses the properties of the supercritical fluid to extract the compound of interest. Basically, the process consists of two fundamental stages: firstly, the compound is extracted by the supercritical fluid and then the fluid is rapidly removed by a change in pressure and/or temperature. Figure 4 shows a schematic representation of SFE equipment with CO_2_ as solvent at a specific pressure and temperature. The extraction process is commonly carried out, combining a static period where the solvent is permanently in contact with the solid, and a dynamic period where the solvent is continually passing through the solid [68].

The extraction conditions that should be optimized to guaranty the maximum efficiency in the extraction by SFE are: temperature, pressure, particle size, amount of co-solvent, moisture content of natural material, time of extraction, flow rate of CO_2_, and L/S ratio. Different studies have been carried out applying SFE to extract flavonoids and anthocyanins in different matrices [68,69,70,71,72,73,74,75]. Table 3 shows some recent examples selected from the literature on the application of SFE in the extraction of anthocyanins from different natural matrices, together with the optimal extraction conditions to achieve maximum yields. Maran et al. [69] observed that at pressures higher than 200 bar, the yields slightly decrease in the extraction of total monomeric anthocyanins in Indian blackberry. They pointed out that temperatures above 50 °C led to a slight decrease in the yield, with the optimum temperature being 50 °C. Furthermore, they demonstrated that the optimum solvent flow rate was set at 2 g/min, showing that higher flow rates had no significant effect on the extraction yield. Other studies, shown in Table 3, have been carried out to optimize these parameters, in order to obtain the maximum yield in the extraction of anthocyanins in different natural matrices. These studies conclude that the recovery of antioxidant compounds improved when the pressure was moderately high (higher than 100 bar), the temperature was moderated (not higher than 100 °C), and the addition of a co-solvent was considered. The amount of co-solvent depends on the time of the extraction and the amount of the powdered sample. The most commonly used co-solvent is ethanol [70]. Jiao et al. [68] compared conventional extraction with SFE with CO_2_ and water as a co-solvent to extract anthocyanins from Haskap berry paste. After optimizing the extraction conditions for SFE (Table 3), they concluded that SFE extraction offers higher anthocyanin extraction efficiency (52.7% vs. 38.3%) and improves antioxidant activity (89.8% vs. 72.2%) compared to conventional extraction [68]. 

Thus, SFE might be considered an effective green technology to extract anthocyanins. One of the main advantages of this extraction method compared with others carried out under low pressure is that the amount of solvent used is greatly smaller [41]. In addition, this technique allows obtaining extract with higher antioxidant activity, and has shown higher efficiency, selectivity, and a lower extraction time [68,71]. Pazir et al., [75] compared the yield of the conventional extraction with the yield of SFE with CO_2_ and ethanol to extract anthocyanins from Merlot red grape pomace. They conclude that, in a short period of time, 80 min, 36% of the anthocyanins could be extracted by SC-CO_2_. However, they failed to obtain recovery percentages similar to those obtained by conventional extraction (63%) when they increased the extraction time to 180 min. However, the increase in the amount of extractable anthocyanins with increasing extraction time corresponded strongly with the increase in total antioxidant activity [75].

In order to improve the efficiency and selectivity of the SFE, different alternatives have been proposed. An alternative is to increase the capacity and selectivity of the supercritical extraction with CO_2_ in the extraction of anthocyanins, based on the application of subcritical CO_2_ extraction. The latter consists of using supercritical CO_2_ followed by supercritical CO_2_ with a certain percentage of ethanol as co-solvent. During the SubC-CO2, the solvent flow through the extraction vessel changes direction, e.g., it may change from an upward to a downward direction. The first and second extraction steps were carried out for 1 h, and the last step for 3 h. In a study carried out by Babova et al. [73] with bilberry, they applied supercritical extraction with CO_2_ followed by subcritical CO_2_ with 10% *v*/*v* ethanol as co-solvent (Table 3). They observed that the subcritical CO_2_ selectively extracted cyanidin-3-O-glucoside and cyanidin-3-O-arabinoside, delphinidin-3-O-glucoside, ellagic acid pentoside, feruloyl hexoside, and several quercetin glycosides. Furthermore, the extract obtained with subcritical CO_2_ extraction showed high antioxidant activity [73]. Other proposals are based on the synergistic effects of combining several extraction techniques. For example, enzyme-assisted supercritical fluid extraction (EASCFE) has been proposed by Mushtaq et al. [76] for extracting antioxidants from pomegranate peel. This approach increases the extraction yield and selectivity, since the enzymatic treatment facilitates the breakdown of the cell wall, allowing the fast penetration of the supercritical fluid and increasing the mass transfer [67]. Another proposal consists of combining ultrasound with SFE. Pasquel Reategui et al. [77] applied this combination to extract the antioxidant form blackberry bagasse. They obtained a higher yield with this combination. This may be because the ultrasound treatment breaks down the cell walls, facilitating the rapid penetration of the supercritical fluid and the extraction of the antioxidants. These proposals increase the efficiency and the selectivity of the extraction, but also decrease the extraction time and, consequently, the extraction costs. These proposals are presented as promising alternatives to conventional techniques for anthocyanin extraction, from the point of view of environmentally responsible extraction technologies. 

Although, a priori, the application of supercritical fluid extraction can be considered too costly to be implemented at a large scale, studies on its viability show that a good optimization of the working parameters (mainly pressure and temperature) allows for obtaining a competitive extraction compared to conventional extraction processes [78]. However, more studies should be performed to optimize other parameters that also have an effect in the efficiency of the extraction. Future studies should focus on the optimization of the extraction time for different geometries of plant materials because the mass transfer could be affected by the nature of the food matrix. Furthermore, the type of co-solvent and the flow rate should be optimized in combination with the extraction time to improve the cost efficiency of the extraction. Finally, further studies, including the economic aspect of extraction are needed to estimate the operating costs of an industrial-scale extraction, accordingly.

##### Pressurized Liquid Extraction (PLE)

PLE, also known as accelerated solvent extraction (ASE), is based on the use of solvents at a temperature between room temperature and 200 °C, and pressure between 35 and 200 bar. These conditions allow bioactive compounds to be extracted relatively easily from various natural matrices. This is due to the fact that, at high pressure, the solvent remains liquid at temperatures above its boiling point, favoring the solubility of the analytes. This extraction technique receives a different name when the solvent used is water. In this case, the technique is known as sub-critical water extraction (SWE). These types of extractions (PLE or SWE) are carried out in an accelerated solvent extractor whose scheme is similar to the one shown in Figure 4.

The SWE method has been used as an economical, green, and sustainable extraction process to extract anthocyanins in different natural matrices. The experimental conditions that should be optimized to obtain the maximum yield of the extraction are temperature, pressure, static time, and number of cycles. Table 4 shows some recent examples selected from the literature on the application of SWE in the extraction of anthocyanins from different natural matrices, together with the optimal extraction conditions to achieve maximum yields. Thus, Wang et al. [79] optimized the pressure, temperature, and time to extract the maximum anthocyanin content from the raspberry by SWE. They concluded that the maximum amount of total anthocyanins (8.15 mg/g) was obtained when SWE was carried out at 70 bar and 130 °C during 90 min. Their results demonstrated that the extraction efficient and the antioxidant activity of anthocyanin obtained from the raspberry by SWE were significantly higher than those obtained by conventional extraction with hot water or methanol [79]. Kang et al. [80] also determined the optimal extraction conditions for extracting anthocyanins from blueberries and chokeberries by SWE. They optimized the temperature (110 °C, 130 °C, 150 °C, 170 °C, 190 °C, and 200 °C), the extraction time (1, 3, 5 and 10 min) and the pH of the solvent (water and 1% citric acid). They concluded that the optimal conditions for blueberries were 130 °C for 3 min and for chokeberries 190 °C for 1 min at a pressure of 100 bar, for both natural matrices. They also concluded that the use of SWE with 1% of citric acid increased the total content of anthocyanins and the content of malvidin-3-galactoside in the extract obtained from blueberries. The total content of anthocyanins and the content of cyanidin-3-galactoside were three times higher in the extract obtained from chokeberries, when 1% of citric acid was used in the solvent. They also concluded that the solubility of anthocyanins depended on their structures; thus, the presence of more methoxy and hydroxyl functional groups in the basic skeleton of anthocyanin will result in a lower solubility [80].

In conclusion, SWE is a green, faster, and more-efficient method to extract anthocyanin than conventional extraction methods. Thus, SWE is presented as a feasible application for the extraction of anthocyanins, and it can be easily implemented on an industrial scale.

PLE has also been applied to increase the extraction yield of different antioxidant compounds, between them, the anthocyanins [22,42,81,82]. The parameters that should be optimized for this technique are the same as for SWE, but in this case, the selection of the solvent is also important. In the case of anthocyanins extraction, acidified aqueous ethanol or methanol are commonly used (Table 4). In relation to temperature and pressure, high pressure and temperature facilitated the penetration of the solvent in the natural matrix. The maximum temperature is limited by the thermal instability of anthocyanins. The static time and the number of cycles should be enough to guaranty the total contact between the solvent and the bioactive compounds in the matrix. Thus, Cai et al. [22] concluded that the maximum amount of anthocyanins from purple sweet potatoes was obtained by PLE using the conditions showed in Table 4, compared with conventional extraction CE and UAE. The extract obtained by PLE contained more diacyl anthocyanins and less nonacyl and monoacyl anthocyanins than CE and UAE extracts. The PLE extract also had higher antioxidant activity than the others measured by FRAP, but not by ORAC [22]. In relation to the number of cycles, Cai et al. [22] and Sabino et al. [42] determined that increasing the number of cycles was negative in anthocyanin recovery. On the other hand, they showed that more than two cycles led to a decrease in anthocyanin content. They justified this effect by the relationship between the number of cycles and temperature. The anthocyanins presented a slight degradation with the increasing of the temperature. However, previous research on PLE has indicated a positive effect of temperature on the extraction of anthocyanins from some vegetal matrices [22,42]. They concluded that the temperature effect on anthocyanin extraction is influenced by the product matrix and the solvent used for extraction. In addition, the high pressure applied in PLE increases the covalent bond stability within molecules, preventing the thermal degradation of these compounds [42].

The results have revealed that PLE allows obtaining antioxidant-rich extracts with high potential application in food, supplements, and pharmaceutical industries [82]. However, it is not free of limitation and some studies have showed lower efficiency than UAE in the extraction of anthocyanins or extract with lower antioxidant activity [22,42]. As a consequence, some alternatives have been studied. Moirangthem et al. [19] proposed the combination of microwave-assisted sub-critical water extraction (MA-SWE), at 90 °C for 5 min, from Manipur black rice. They concluded that this combination allowed obtaining an extraction efficiency of 85.8% and extracts with higher antioxidant activity than an equivalent conventional extraction with methanol. They consider that a validation at a small-scale level is needed to assess its economic feasibility of MA-SWE [19]. Andrade et al. [83] proposed another alternative consisting of combining ultrasound-assisted with pressurized liquid extraction (US-PLE) to improve the yield of anthocyanin extraction from black chokeberry pomace. They concluded that the effects of pressure and sonication were more pronounced at low temperatures and using slightly acidified solvent, lower than 1.5%. Thus, US-PLE showed clear advantages reaching a high total extraction yield. However, an economic assessment is necessary in order to evaluate the influence of the capital investment on the process profitability at an industrial level [83].

Thus, the PLE method is an emerging technique that carries out fast and efficient extractions under high temperature and pressure conditions. The association of green technologies with green solvents makes PLE an eco-friendly technology. The PLE allowed obtaining antioxidant-rich extracts with high application potential in food, supplements, and pharmaceutical industries. In comparison to conventional methods, it affords better results for the recovery of these compounds, since the viscosity of the solvent decreased, favoring the solubilization of the compounds of interest. This allowed high penetration of the liquid solvent in the solid matrix (raw material) and increased the diffusion and mass transfer coefficient, improving the extraction performance. However, the negative effect of temperature on the extract of some natural matrices may lead the industry to opt for other techniques, such as UAE rather than PLE or SWE.

##### High-Pressure Liquid Extraction (HPLE)

**Figure 5:**
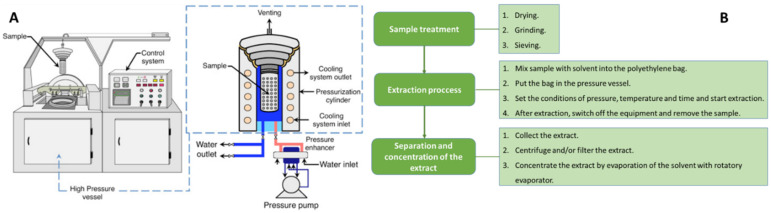
(**A**) Schematic representation of high-pressure processing equipment. (**B**) Schematic procedures of UHPE processing. ((**A**) taken from Barba et al. [85], with permission from Elsevier).

HPLE includes techniques known as high hydrostatic pressure extraction (HHPE), ultra-high pressure extraction (UHPE), and high-pressure processing extraction (HPPE). They are characterized as using higher pressure than PLE in order to keep the solvent beyond its boiling point. This further facilitates the extraction and decreases the amount of solvent used and the extraction time [32]. The latter advantage is directly related to the lower exposure of the sample to high temperatures, which favors the quality of the extract. These techniques are some of the most recent extraction techniques developed to increase extraction yields, by applying non-thermal green technology. These techniques use pressures from 100 to 800 MPa or even more than 1000 MPa [33,41], allowing for better penetration of the solvent into the cell membrane, improving the bioaccessibility. Studies have demonstrated that the higher the hydrostatic pressure, the more components can be released and higher yield of extraction can be obtained [41,84]. The main disadvantage of these extraction methods is the cost of the energy needed to obtain the higher pressures. Currently, the limit of the high pressure at the industrial level is 600 MPa [41]. Figure 5 shows a schematic representation of high-pressure processing equipment. This equipment basically consists of a high-pressure vessel, a high-pressure pump, and a cooling system. This figure also shows a scheme on the different steps that should be followed to perform UHPE. 

In order to guaranty the maximum efficiency of the extraction, the factors that should be optimized are the composition of the solvent, pressure, temperature, particle size, moisture content of natural material, time of extraction, and solvent-to-solid ratio. The most important of these factors are solvent, pressure, temperature, and L/S ratio. Martin and Asuero [33] recently published a table with selected applications of HHPE for the recovery and purification of anthocyanins in fruits, vegetables, and juices [33].

Table 5 shows some current examples of the optimal experimental conditions applied to extract anthocyanins from different natural matrices by HHPE. Fernandes et al. [86] carried out a study to optimize the pressure (0–500 MPa), time (5–15 min), and ethanol concentration (0–100%) to extract flavonoids, tannins, and anthocyanins from dried pansies by HPLE. They concluded that the optimum conditions were to apply a pressure of 384 MPa for 15 min with 35% (*v*/*v*) ethanol as solvent. They determined that a mixture of water and ethanol (20–70% *v*/*v*) as solvent increased the values of monomeric anthocyanins compared with only the water of ethanol as solvent. They also observed that high pressure increases the values of monomeric anthocyanins. It could be explained by the fact that high pressure has the ability to reduce the pH value of the solvent during extraction, derived from the enhanced protonation of molecules present in the extract [86]. This might increase the extraction effectiveness of anthocyanins, stable at acid pH. In addition, they determined that anthocyanins are selectively extracted depending on pressure intensity. A pressure at 200 MPa favors the extraction of anthocyanin monoglucosides, whereas a pressure at 600 MPa increases the extraction of acylglucosides [86]. In relation to the extraction time, they concluded that it is not a relevant factor in the amount of anthocyanins extracted, since the high pressures used make the permeabilization of the cell almost instantaneous [86].

Briones-Labarca et al. [87] demonstrated that the application of HHPE to extract the bioactive compound from discarded blueberries increased the content of anthocyanins, polyphenols, and flavonoids, as well as the antioxidant capacity by DPPH and FRAP compared with conventional extraction. However, although the bio-accessibility of the polyphenols and flavonoids also increased with HHPE vs CE, the anthocyanins showed a decrease in the bio-accessibility after HHPE. In addition, they proved that the anthocyanins extracted increased from 15.1 to 39.4% with the time of the extraction from 5 to 15 min when compared to CE [87].

Compared to conventional techniques, HHPE uses less solvent volume, shorter times, and increases extractability, antioxidant capacity, and bio-accessibility of the bioactive compounds present in the natural matrices. In general, bioactive compounds remain unaffected by the pressure, while the structure of large molecules can be altered by high pressure [33]. Under UHP conditions, the differential pressure between the inside and outside of the cell is very large. This is why the solvent permeates very quickly through the ruptured membranes in the cells, increasing the mass transfer rate of solute. This could result in a very short extracting time of UHPE. The holding time should also be carefully considered because too long of a pressure holding time can damage the biological activity of extracts [41]. Pressures above 400 Mpa present the advantage that the activity of some oxidative enzymes, such as polyphenol oxidase (PPO) and peroxidase (POD), decreases, and the activity of the superoxide dismutase remains unaltered [88]. Temperature is the other important factor to control during HPLE because it directly affects the efficiency and selectivity during extraction. In general, HPLE does not use heat as an energy source. HPLE is usually carried out under refrigeration or at room temperature (Figure 5, Table 5) helping to protect thermosensitive biocompounds, such as anthocyanins. However, high temperatures (50–100 °C) improve the efficiency of the extraction because the diffusion rate is incremented, allowing for faster extractions. Thus, the choice of extraction temperature depends on the stability of the compounds and the extraction yields required. In the extraction of thermolabile compounds, high temperatures may cause the degradation of extracts [34]. However, it has been shown that, depending on the pressure and time conditions applied, some microorganisms could not be inactivated. In order to avoid this problem, some authors suggest combining HHPE with temperature, which increases the efficiency of the extraction and decreases the enzymatic action and the antimicrobial effect, preserving the antioxidant activity. Thus, the combination of HHPE and temperature for a short time of processing produce high nutritional and sensory qualities in the food [33,89,90,91,92]. Additionally, the high-pressure effect on anthocyanin content cannot be generalized, because of the composition of the matrix, the activity of the oxidative enzymes, the pressure and holding time could compromise the efficiency of the extraction. For that reason, it is recommended to not only optimize the extraction conditions to increase the amount of anthocyanins extracted, but to also carry out a bioaccessibility evaluation from bioactive compounds extracted by HHPE. The high pressure applied during HHPE could affect the conformation and the structure of macromolecules and also provoke the formation of metabolites that may exert biological action on the extract. Therefore, a bioaccessibility study of the extract is recommended.

The application of HPLE on an industrial scale is limited by the cost of the equipment. On the other hand, the technology is currently only available for batch processes, which also implies higher costs.

#### 2.2.4. Pulsed Electric Field Extraction (PEFE)

**Figure 6:**
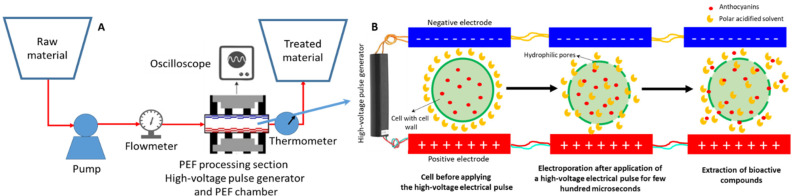
(**A**) Schematic diagram of a high-intensity pulsed electric field (PEF) continuous extraction. (**B**) Electroporation mechanism for bioactive compound extraction.

PEFE is another novel, environmentally friendly cell membrane permeabilization technique that breaks down the cell wall of plants using electrical pulses. Different studies have showed that the increment of the temperature during the application of high-voltage electrical pulse is not higher than 10 °C [41,93]. Thus, this technique can also be classified as a non-thermal extraction method, which makes it an ideal technique to extract thermolabile bioactive compounds, such as anthocyanins. The pulsed electric field technique is based on the application of short duration pulses of moderate to high electric field strengths ranging from 0.1 to 0.3 kV/cm in batch mode and 20–80 kV/cm in continuous mode extraction at room temperature [35]. The basic principles of PEFE processing are based on innovative concepts within electrical engineering, fluid mechanics, and biology. The application of PEFE to biological cells is based on the principle of electropermeabilization, due to an induced transmembrane potential. The electroporation or electropermeabilization process enhances the permeability of cell membranes by an external electrical force [13,14,94]. The biological sample is placed between the electrodes and a high-voltage electrical pulse is applied from a few to several hundred microseconds. The electric field generated is able to induce the formation of hydrophilic pores in the cell membrane, which opens protein channels (Figure 6). This process is known as “electroporation” [35,93]. The strong electric field applied on the cell sample originates accumulation of oppositely charged ions on both sides of the membrane. When transmembrane potential exceeds a critical value of about 1 V, the repulsion between charge-carrying molecules leads to membrane thickness reduction and permeabilization to small molecules [95]. The sample experiences a force per unit of charge called “the electric field” when high-voltage electrical pulses are applied through the electrodes. Based on this process, the membrane cell loses its structural functionality, and the subsequent extraction of the bioactive compounds can be easily performed [35]. Depending on electric field strength and treatment intensity, the permeabilization might be reversible or irreversible. To achieve good extraction results in soft plant tissues, electric field strengths should be applied between 0.1 and 10 kV/cm. However, electric field strengths up to 20 kV/cm should be applied in order to achieve good extraction results from seeds and stalks where lignification can occur [95]. Figure 6 shows a schematic diagram of a high-intensity pulsed electric field (PEF) continuous extraction system together with the electroporation mechanism for the extraction.

Different parameters should be optimized to improve the extraction efficiency of bioactive compounds applying PEFE. The most important parameters are the exposition time, the electric field strength, and the total specific energy input of the pulse. Some authors have demonstrated that the size of the chamber also affect the yield of the extraction. Thus, a chamber with a larger diameter allows the application of a higher number of pulses and increases the residence time. In addition, a large diameter chamber would allow to work in a continuous mode [96]. Another factor that affects the yield of the extraction is the initial temperature of the process. Thus, Gagneten et al. [97] evaluated the effect of the initial temperature at 10 or 22 °C. They observed that the efficiency of the extraction was higher at 22 °C. The temperature at 22 °C was associated with a higher electric current than the temperature at 10 °C. In addition, they observed that during the extraction the temperature increased 5 °C in both experiments and the correlation with the electric current was linear. Thus, cold temperatures can provoke a decrease in the electroporation efficiency [97]. This technique has been applied to improve the extraction efficiency of anthocyanins in different natural matrices. Table 6 shows some current examples of the optimal extraction condition used for different natural matrices and the yield obtained by PEFE. Lamanauskas et al. [98] concluded that the best conditions to optimize the extraction of anthocyanins from European blueberries were to expose the sample for 20 μs to monopolar square wave pulses with an electric field strength of 5 kV/cm and a total specific energy input of 10 kJ/kg. They found that an increase in the pulse electric field intensity (strength and total energy) resulted in a significant increase in the rate of cell disintegration. Under these conditions, they managed to increase the anthocyanin extraction yield by 8.3% compared to the extraction without applying PEFE [97]. Taiebirad et al. [99] optimized the extraction condition to extract the maximum yield of anthocyanins, to improve the antioxidant capacity by DPPH and to increase the extraction of the bioactive compounds for barberry fruit by PEFE. They applied an intermittent electric field at three levels of electric field intensity (0.5, 1.75, and 3 kV/cm) and three levels of number of pulses (15, 30, and 45). They observed that the total amount of anthocyanins extracted increased when the number of pulses and the intensity of the pulse increased, in agreement with the results obtained by Lamanauskas et al. [98]. However, they noted that the increase of the electric field strength and the number of pulses initially increased the flavonoid content and the DPPH capacity, but with the increase of these variables, these two properties decreased. A study carried out by Lončarić et al. [95] corroborated these results. They compared high voltage electrical discharges (HVED) PEFE and UAE, in terms of extraction yield of total and individual phenolic acids, anthocyanins, and flavanols of blueberry pomace extracts. The highest total content of anthocyanin (1757.32 μg/g of DW) was obtained in the methanol-based solvent by PEF-assisted extraction after 100 pulses and 20 kV/cm and at energy input of 41.03 kJ/kg. However, the other antioxidant compounds were extracted better in ethanol-based solvent. Other studies observed no significant effects in the anthocyanin yield when they increased the electric field intensity. Aadil et al. [100] investigated the effect of PEFE with different electric field strengths: 0, 5, 10, 15, 20, and 25 kV/cm in grapefruit juice. They observed no significant change in pH, Brix, titratable acidity, sugars, total anthocyanins, and color attributes with the increase in pulsed electric strength as compared to control treatment. However, a significant decrease in non-enzymatic browning (NEB), viscosity, and in the activity of microorganisms was observed. In addition, they realized an increase in cloud value, DPPH, total antioxidant activity, total phenolic compounds, and total carotenoids with the increase of pulsed electric strength compared with the control treatment. Finally, the authors suggest that applying PEFE at 25 kV cm^−1^ could improve the quality of grapefruit juice, although the anthocyanin content would not be significantly increased. These results are in agreement with those obtained by other authors [27,98,99,101]. 

The combination of PEFE and UAE was explored in order to obtain higher anthocyanin extraction yields and to increase the quality and the antioxidant capacity of the extracts (Table 6) [102,103,104]. A study carried out by Barba et al. [105] on the extraction of anthocyanins from grape pomace showed that PEF treatment allowed a selective recovery of extracts with an amount of anthocyanins—22 and 55% higher than with UAE and HVED, respectively. Thus, they suggested that a combination of different extraction techniques, such as PEF + HVED or PEF + supplementary extraction + HVED, is a good alternative to use in the food industry, since it allows, in the first step, an extraction of sensitive compounds, such as anthocyanins, and in the second step, more resistant compounds, such as phenols and flavones [105]. In conclusion, it can be noted that the electric field strength plays an important role in the selectivity of the extraction. This conclusion reveals the complexity of the optimization of the different parameters, and the usefulness of statistical treatments to determine the optimal conditions depending on the extraction objective [103]. 

One of the main advantages of PEFE, EUS, and HPLE, is the ability to inactivate the microbiota present in food, ensuring food safety, while increasing food quality [111]. The application of pulse electric field (PEF) in the industry, for the purpose of pasteurization, has been extensively investigated, with PEF-assisted sterilization achieved under laboratory conditions [94,112,113]. However, some studies have shown that PEF processing requires more energy and is consequently more expensive than thermal processing [94,111]. This disadvantage could be reduced by improving the processing chamber to reduce inactivation areas, promoting a more homogeneous treatment, and by improving experimental planning [94]. In addition, the advantages of PEF could motivate the industry to implement this technique, despite this disadvantage. The main advantages of PEF are: it is a sustainable technique due to the non-thermal treatment, its beneficial effects on food quality (by increasing the content of bioactive compounds), and the ability to selectively inactivate microorganisms.

Yildiz et al. [102] compared different extraction techniques with microbiota inhibition capacity (HPLE, USE, and PEFE), with a conventional thermal pasteurization treatment. Their objective was to study the effect of these treatments in the quality and antioxidant properties of strawberry juice. All techniques significantly reduced the initial natural microbiota. However, they pointed out that HPLE and PEFE obtained juice with significantly higher amounts of total anthocyanin content 15–17% in comparison with untreated strawberry juice. Furthermore, they observed higher radical scavenging activity in the juice extracted by HPLE and PEFE than in the juice treated with thermal pasteurization. A statistical analysis indicated that HPLE and PEFE extracted juices with similar antioxidants content and antioxidant activity. Both techniques obtained better extracts than the extract obtained by sonication, thermal pasteurization, or untreated. The study concluded that HPLE and PEFE produce enhanced quality fruit juices [102]. Even better results were obtained when more than one extraction technique was combined. Thus, Faisal Manzoor et al. [104] investigated the combined effects of the ultrasound (US) and pulsed electric field (PEF) treatment on spinach juice. They demonstrated that both techniques provide spinach juice with an acceptable higher quality than the juice untreated. However, their results revealed that the combined treatment based on US-PEF increased the concentration of flavonoids, phenolic, flavonols, anthocyanins (Table 6), carotenoids, total chlorophyll, vitamin C, DPPH activity, and the total antioxidant capacity in the spinach juice than single treatments or the untreated sample. Furthermore, this combination of techniques that preserves antioxidants and inactivates spoilage enzymes was better than the individual treatments or the untreated sample. This study confirms that US-PEF can enhance the quality of spinach juice at an industrial scale [104]. The results indicate that the combined treatments of non-thermal technologies are useful for the food processing and preservation industries, but they are slightly costlier than using individual treatments. However, the combination of non-thermal techniques allows for high quality, healthy, and safe food to be produced, covering the demands of the consumers, although it is likely to be marketed at higher prices to cover production costs.

Additionally, to the combination of PEFE with UAE, or another extraction technique described above, other electrical techniques have recently been proposed to improve the extraction of bioactive compounds. These techniques are known as high-voltage electrostatic fields (HVEFs) and high-voltage electric discharges (HVEDs). 

**HVEF** is a non-pulsed electrostatic field treatment. This technique can also modify the structure of animal and vegetable tissues favoring the extraction of valuable components from different materials (e.g., anthocyanins). As in the case of PEF, this technique is used for the extraction of bioactive compounds and to inactivate microorganisms and enzymes in liquid matrices, by means of a non-thermal treatment, while preserving the nutritional quality of the food [21].

**HVED** is based on both chemical reactions and physical processes. When a HVED is produced directly in water, it injects energy directly into an aqueous solution through a plasma channel produced by a high-current/high-voltage electrical discharge (>40 kV; >10 kA) between two submerged electrodes. The action of HVED is based on two phases: the pre-breakdown phase and the breakdown phase. During the first phase, relative weak shock waves are produced and, as a result, it the formation of a few little bubbles can be observed. Furthermore, strong UV radiations and active radicals are generated. These phenomena cause cell structure damage and accelerate extraction of intracellular compounds. Thus, it is important to control the electric field intensity to avoid the damage of the bioactive compounds and only motivate the wall cell destruction. The enhanced electrohydraulic phase occurs during the transition of pre-breakdown to breakdown phase, having several effects: strong shock waves, strong UV radiations (200–400 nm), production of highly concentrated free radicals, bubbles with plasma inside and strong liquid turbulence. They provoke mechanical destruction of cell tissues and oxidation, which could affect the antioxidant activity of bioactive compounds [95]. In general, this extraction provides a more powerful mechanical disintegration of the cell walls and, consequently, it allows achieving a more effective extraction. Thus, this technique has been applied in removing organic impurities of water [20], improving the shelf life and the antioxidant capacity of fruits [114], exploring its utility in microbial and enzymatic inactivation, winemaking, and thawing, drying, and freezing in different foods, among others [115]. This technique has also been used to increase the extraction yield of bioactive compounds from different raw materials. Thus, Lončarić et al. [26] carried out a study to evaluate the capacity of HVED to extract anthocyanins, including malvidin, delphinidin, peonidin-3-O-glucoside, and cyanidin-3-O-glucoside from indigenous fungus-resistant grape by-products compared to UAS. They concluded that the extraction carried out by HVED showed high yields of all analyzed compounds compared to UAS [26]. 

Studies have demonstrated the efficacy of HVED treatment in the aqueous extraction of bioactive compounds, in particular polyphenols from different matrices. [116,117]. Thus, Rajha et al. [117] studied the mechanical, electrical, and chemical effects of HVED and their roles in polyphenol extraction from vine shoots. This study demonstrates the efficacy of electrical discharges for the intensification of the extraction of bioactive molecules (polyphenols) from a specific byproduct (vine shoots). It also shows HVED as an energy-saving extraction process, relevant for industrial application, since no organic solvents were used, and the process duration and temperature were reduced. However, HVED also presents some disadvantages. The intense cell wall rupturing power of HVED extraction could cause the formation of small particles, which may hinder the subsequent separation stage [116].

#### 2.2.5. Enzyme-Assisted Aqueous Extraction (EAE)

Another strategy to access the interior of cells and extract bioactive compounds is based on a biological technology, which involves the use of enzymes. Enzyme-assisted aqueous extraction (EAE) has been used for years and has improved over time. This technique has the following advantages: (i) the enzymes are highly selective and efficient; (ii) they work under moderate conditions of pressure and temperature; (iii) some have the properties to destroy or degrade the cell wall; and (iv) it can be qualified as a green technique [41,118,119,120,121]. Today, advances in enzyme catalysis, the availability and diversity of enzymes, as well as increasing environmental constraints, make this technology a potential tool for industrial application. 

Enzymes can be used as their own extraction methods by EAE or as additional tools to other extraction processes to increase yields of extraction by weakening the vegetal cell walls. The principle of the enzymatic extraction is based on the action of the enzyme on the wall cells. In order to reach the bioactive compounds stocked in vegetal cells, several barriers have to be crossed: extracellular cell walls, cell walls, and oleosomes. Each of these barriers is composed of its own constituents. They are naturally synthesized and hydrolyzed by specific enzymes. The most common enzymes used to hydrolyze part of the constituents of vegetal cell walls could be group in four different families: cellulases, hemicelluloses, pectinases, and proteases. The three first families can be used alone or in combination, each one having a different effect in the cell wall. Thus, a selective extraction of different bioactive compounds can be achieved according to the enzyme applied [118]. Cellulases, hemicelluloses, pectinases, and proteases have been employed to enhance and accelerate pigment extraction of various plant materials, being able to extract pigments quicker and with higher efficiencies than conventional ethanol extraction [119]. However, proteases have to be used separately from the other families of enzymes because they are able to hydrolyze the enzymatic proteins, decreasing or eliminating the specific activities of the enzyme mixture. The enzymes applied in EAE can be obtained from different natural sources, such as bacteria, fungi, vegetable, and fruit extracts, or animal organs [41]. Depending on the source of the enzyme, they have different properties and act under different conditions. Thus, in general, an animal enzyme has a denaturing temperature of 40–45 °C, whereas this temperature is more than 60–65 °C for enzymes produced by microorganisms. Nevertheless, some enzymes are thermo-resistant and could tolerate more than 100 °C during several minutes. Currently, different commercial enzymes can be acquired in the market.

EAE has been applied to extract pigment, phenols, and anthocyanins in different natural matrices, improving the yield of the extraction in comparison with the conventional techniques [118,119,120,121]. Table 7 shows some current examples of the optimal extraction conditions used for different natural matrices and the yields obtained by EAE. The main extraction conditions that should be optimized to increase the yields of the extraction by EAE are: the composition of the enzymatic mixture, pH, temperature, L/S ratio, enzymes/solid ratio, and hydrolysis time. 

The composition of the enzymatic mixture is a key factor. With a high amount of different enzymes, the yield of extraction will be high and rapidly achieved. However, the optimal enzymatic mixture must be adapted to each kind of matrix and each bioactive compound [118,122]. In some cases, when more than one compound is to be extracted, a multistep enzymatic extraction is applied. Thus, it is necessary to optimize each extraction condition, such as pH or temperature, for the hydrolysis of the walls, according to each enzyme.

The pH is another important factor to be optimized for several reasons: (i) an acid pH is important to keep the structure of the anthocyanins stable; (ii) an acid pH provides an increase in wall plasticity and intermolecular sliding; (iii) the enzyme activity (ionization) depends on the acid pH, thus, an inappropriate pH might strongly decrease the enzymatic activity. In addition, pH-related denaturation may be irreversible to some enzymes. In consequence, when an enzymatic mixture is used, in order to promote the optimum enzymatic hydrolysis for each enzyme, it is necessary to define an average pH that covers the optimum pH for each enzyme in the mixture.

As with pH, temperature is an important, because it has an effect in the viscosity of the matrix: a high temperature reduces the viscosity and facilitates the extraction. However, the stability of the extract could also be affected by temperature, decreasing if the temperature is too high. In addition, the enzymatic activity depends on the temperature. Catalytic activity of the enzyme is higher with an increasing temperature. However, since they are also proteins, thermal denaturing is possible with a heating step. Thus, the temperature reaction is an important factor that should be kept in mind for enzymatic extraction. 

As in other extraction techniques, the optimal liquid/solid ratio is a parameter often discussed in the literature [119,120]. It is different, depending on the studied matrix; often, it is even different for the same matrix. Sometimes the L/S ratio is defined according to the equipment and the system for stirring available. 

Likely, to the L/S ratio, the enzyme/solid ratio and hydrolysis time are discussed. In theory, hydrolysis time and enzyme concentration are well correlated. For example, the hydrolysis time can be halved by doubling the enzyme concentration, without saturating the mixture, giving similar results in the extract. In practice, the effectiveness of the enzymes decreases more-or-less quickly when the hydrolysis time is prolonged. Different inhibition factors may occur: from the matrix, from products of reactions, from the process factors, etc. Thus, some studies have proven that an increase in the enzyme dose raises the recovery rate of total anthocyanins until the middle of the experiment, and afterwards starts to decline. The negative effects of the higher enzyme dose could indicate that the preparations might possess secondary enzyme activities that catalyze the degradation of anthocyanins [119,121].

More recently, some improvements of this technique have been introduced in order to enhance anthocyanin extraction yield. Thus, Xue et al. [129] suggested combining the power of enzymatic treatment with the power of ultrasonic extraction by a new extraction technique known as ultrasound-assisted enzymatic extraction UAEE. They applied this combination to extract anthocyanins from raspberry wine residues. In this study, under the optimal conditions (Table 7), the anthocyanin yield and the extraction efficiency using UAEE were higher, 0.853 mg/g and 84.75%, respectively than those obtained by the other three conventional extraction methods (hot water extraction, acidified ethanol extraction, and EAE). In addition, they determined that the extract obtained by UAEE presented high activities of DPPH 417.15 Trolox equivalents/g extract and ABTS 520.07 Trolox equivalents/g extract, reducing power (412.79 Trolox equivalents/g extract). Thus, UAEE allows a higher yield with less energy consumption and better selectivity than the conventional extraction methods. They concluded that the UAEE of the desired anthocyanin components from natural plant resources is a rapid, efficient, and environmentally friendly extraction method [129]. Similar conclusions were reported by Oancea and Perju [128], who obtained high amounts of total anthocyanins (676.03 mg/100 g DW) and strong antioxidant activity in the crude acidified hydroethanolic extracts obtained by UAEE with cellulases.

EAE and its different combinations, such as UAEE, improve the yields of anthocyanin extraction and have a number of advantages mentioned above. However, it has a number of drawbacks that limits, in part, its use on an industrial scale. The use of enzymes has some commercial and technical limitations that should be considered. Enzyme cost and process energy are high, and enzyme activity is highly dependent on pH, temperature, and nutrient availability, as different combinations of enzyme preparations need to be tested and optimized. These factors influence the application of the EAE; thus, further research will be required to improve its application at an industrial scale [41,120].

## 3. Advantages and Disadvantages of the Promising Green Extraction Techniques

According to the results presented above, the most promising green techniques to improve the anthocyanin yield and the extraction efficiency from natural matrices have been presented. These techniques are considered as ‘green non-conventional techniques’. All of them have the following in common: they reduce processing time, temperature, energy consumption, and the use of organic solvents, in comparison with conventional techniques. In addition, they have previously been shown to be effective in the extraction of anthocyanins; thus, which of these techniques is the most suitable for extracting anthocyanins at an industrial scale? It is not possible to give a clear answer to this question, as each technique has a number of advantages and disadvantages. In general, the selection of extraction methods depends mainly on many factors, such as the physicochemical properties of the compound and solvent, the economic value of the compound, environmental concerns, the cost of the process, the required instrumentation, among others. Table 8 shows a comparison between these techniques based on their strengths, weaknesses, and suitability to extract anthocyanins. 

These techniques have been used not only in isolation to extract anthocyanins, but their combined use has also been explored to improve the extraction of anthocyanins. In general, this alternative produces a more effective extraction than the use of a single one, as is the case of UAE combined with microwave UMAE [49], enzyme-assisted supercritical fluid extraction (EASCFE) [76,77], microwave-assisted sub-critical water extraction (MA-SWE) [19], ultrasound-assisted with pressurized liquid extraction (US-PLE) [83], ultrasound and pulsed electric field US-PEF [104], or ultrasound-assisted enzymatic extraction (UAEE) [128,129]. In some cases, the combination of these techniques could provoke deterioration of the extract. In order to avoid the deterioration of the extract during the process, a specific optimization of the extraction parameters should be done according to the matrix and the extracted compounds. In addition, innovative approaches are needed for overcome these shortcomings. Furthermore, the combination of various techniques implies even greater complexity for large-scale implementation. The scaling of the extraction methods to industrial scale presents some disadvantages, such as insufficient recovery, degradation due to excessive heating and extraction time, which ultimately results in high-energy consumption [29]. To choose a suitable extraction process for anthocyanins it is necessary to consider the extraction efficiency, economic feasibility, and environment aspects. Extraction efficiency of non-conventional extraction techniques is clearly advantageous compared with the conventional extraction methods in regard to time, energy, and extraction yield. Nevertheless, the extraction efficiencies among non-conventional extraction techniques are different (Table 8). For these reasons, nowadays, conventional techniques are still used at the industrial scale [39].

## 4. Conclusions

Extraction techniques trends have evolved, as more advanced techniques making use of green extraction concepts have emerged. The advantages of such techniques include producing good quality yield, lesser solvents and energy consumption, and shorter extraction times. Scientific literature shows clear evidence that extraction procedures of target compounds from natural matrices must be assessed individually. In order to take full advantage of the technological advances in the extraction techniques, the extraction conditions need to be optimized. Mathematical solutions could increase the efficiency and profitability of the process and help to change conventional extraction approaches. 

The selection of an extraction technique is not an easy task as documented above. It is necessary to take multiple factors into consideration: (i) the cost of the instrumentation, maintenance, material, and process. It is necessary to find a compromise between the amount of energy or solvent consumed and the time of the extraction; (ii) yield is another economic factor, but not the only one. The reaction must be sufficiently effective to give good yields and quality of molecules of interest in this case anthocyanins; (iii) microbiology: a mixture of water and organic matter is a very favorable environment for the development of microorganism. The time of the extraction and the conditions applied must consider this factor; (iv) product quality: some products may be sensitive to oxidation or temperature. Extraction for too long, or with too much energy, may affect the quality of the extract or the concentration of antioxidant to be extracted.

In order to consider all these factors, it is necessary to design an extraction process to optimize the extraction conditions. The steps to be taken in such a design are as follows: (i) adjusting extraction conditions (solvent choice, temperature, particle size of sample, pressure, extraction time, among others) according to the characteristics of anthocyanins and natural matrices. The selection is important for achieving high extraction efficiency with high quality extracts; (ii) it is also important to study the influence of the extraction conditions in the bioactivity of the extract. It should be investigated independently; (iii) once that extraction conditions with significant impact in the extraction are selected, the optimization of them with the minimum experimental trials should be done. Commonly, screening experimental designs and optimization experimental designs are used. Screening designs can be used to analyze the most important conditions and their interactions from all potential conditions. The most common design used in the literature for the screening purpose are: two-level full factorial, two-level factorial and Plackett–Burman design. For optimization designs—central composite design (CCD), Box–Behnken design (BBD), Taguchi design, and Doehlert design are used.

Although design processes are applied, advanced extraction methods have limitations in scaling up for pilot or industrial purposes. Value changes and experimental conditions in the laboratory are mostly not optimized for industrial use. Therefore, the development of economically viable industrial extraction methods and tools for mass extraction are needed. In addition, they are limited for industrial applications due to the high equipment costs and complicated installation procedures. Thus, establishing the balance between “energetic” and cost will be a key focus of research in the future. To take advantage of the different extraction methods and to limit their drawbacks, the combinative applications of multiple extraction technologies and the automated potential of these non-conventional extraction technologies would be development tendencies in the near future.

## Figures and Tables

**Table 1:** Current examples of the application of UAE for the extraction of anthocyanins from different natural matrices. Optimal extraction conditions to achieve maximum yield.

Natural Matrices	Tª (°C)	Solvent (%)	Time (min)	L/S Ratio (mL/g)	Power (W)/ Frequency (kHz)/Solid Amount (g)	**Steps after Separation.** **Comments on the Extract.**	Recovery	Ref.
**Jabuticaba epicarp.**	30–35	Ethanol 34.47%	24.44	100:5	500/20/2.5	Centrifugation. No selectivity in extraction was verified.	32 mg of D3G + C3G/g of extract.	[11]
**Purple sweet potato.**	60	Ethanol 90% (0.1% HCl)	60	100:5	200/-/10	Centrifugation and evaporation. Content of nonacyl and monoacyl anthocyanins higher than diacyl anthocyanins in the UAE extract.	214.92 mg of C3GE/100 g of potato DW.	[22]
**Red cabbage.**	40	Ethanol 42.39%	75	3:1	-/37/-	Filtration, vacuum filtration, and evaporation. No selectivity studies. The UAE extract has 12% more anthocyanins than the CE extract.	58.67 mg of C3G/L of extract.	[37]
**Fig *(Ficus carica* L.) peel.**	30–35	Ethanol 100%	21	100:15	310/-/2.5	Centrifugation filtration, and evaporation. Purity of the extract: 9.1 mg of C3R/g of extracted residue. This results were better than the obtained by MAE.	4.32 mg C3R/g of fig peel DW.	[38]
**Jambolan (*Syzygium cumini* L.) fruit.**	30	Ethanol 79.6%	7.5	15:1	Power density: 112.5 W/L/40/4	Filtration and evaporation. Ethanolic extract obtained by BUE was rich in D3,5DG, Pt3, 5DG, and M3,5DG.	(BUE) 54.2 mg C3GE/g of fruit DW.	[42]
**Jambolan (*Syzygium cumini* L.) fruit.**	30	Ethanol 79.6%	7.5	15:1	Power density: 5000 W/L/20/4	Filtration and evaporation. Ethanolic extract obtained by PUE was rich in diglucosides of delphinidin, petunidin, and malvidin. This extract was richer in these anthocyanins than the extract obtained by BUE by 15–25%.	(PUE) 60.5 mg C3GE/g of fruit DW.	[42]
**Red cabbage.**	30	Water	15	100:2	100/30/2	Filtration and centrifugation. No selectivity studies.	20.9 mg of P3G/L of extract.	[43]
**Black carrot pomace.**	50	Water	20	3:1	102/24/75	Centrifugation. Study of the extraction yield of individual anthocyanin, two non-acylated anthocyanins, and three monoacylated anthocyanins in black carrot.	12.4 mg of C3XGG/L of extract. 69.7 mg of C3XG/L of extract. 16.0 mg of C3XGGS/L of extract. 73.4 mg of C3XGGF/L of extract. 34.2 mg of C3XGGC/L of extract.	[44]
**Mulberry (*Morus nigra*) pulps.**	48	Methanol 76% pH = 3	10	12:1.5	200/24/1.5	Filtration and dilution with the same solvent. No selectivity studies.	149.95 μg of C3G + C3R + C3MG + C3DG/g of mulberry FW.	[45]
**Haskap (*Lonicera caerulea* L.) berries.**	35	Ethanol 80%, (0.5% formic acid)	20	25:1	100/40/-	Centrifugation and filtration. C3,5DG, C3G, C3R, P3G, PE3G, were identify in the extract. The anthocyanin/phenolic ratio in the extract was from 62.5 to 92.19%.	22.45 mg C3GE/g of berries DW.	[46]
**Blueberries (*V. Angustifolium* Aiton).**	65	Ethanol 60% acidified	11.5	50:1	100/40/-	Centrifugation and filtration. No selectivity studies.	13.22 mg C3GE/g of blueberries DW.	[47]
**Blackthorn *(Prunus spinosa* L.) Fruit Epicarp.**	Room Tª	Ethanol 47.98% acidified (citric acid, pH = 3).	5	100:5	400/40/2.5	Centrifugation, filtration, and drying. Purity of the extract: 18.17 mg of C3R + P3R/g of extracted residue.	11.76 mg of C3R+P3R/g of fruit epicarp DW.	[48]
***Purple Majesty* potato.**	33	Ethanol 70%	5	200:5	35/20/5	Filtration. Better yields were obtained from raw freeze dried potato than from microwaved or raw sliced potato.	364.3 mg C3G/kg of potato FW.	[49]

Note: CE, conventional extraction; C3DG, cyanidin-3-O-(6″-dioxalyl-glucoside); C3,5DG, cyanidin 3,5-diglucoside; C3G, cyanidin-3-O-glucoside; C3GE, cyaniding 3-glucoside equivalents; C3MG, cyanidin-3-O-(6″-malonyl-glucoside) C3R, cyaniding-3-O-rutinoside; C3XG, cyanidin-3-xyloside-galactoside; C3XGG, cyanidin-3-xylosyl-glucosyl-galactoside; C3XGGC, cyanidin-3-xylosyl-glucosyl-galactoside-coumaric acid; C3XGGF, cyanidin-3-xyloside-galactoside-glucoside-ferulic acid; C3XGGS, cyaniding-3-xylosyl-glucosyl-galactosidesinapic acid; D3,5DG, delphinidin-3,5-diglucoside; D3G, Delphinidin-3-O-glucoside; DW, dried weight; FW, fresh weight; M3,5DG malvidin-3,5-diglucoside; PE3G, peonidin 3-glucoside; P3G, pelargonidin-3-glucoside; P3R, peonidin 3-rutinoside; Pt3, 5DG, petunidin-3,5-diglucoside; Ref. reference; Tª, temperature.

**Table 2:** Current examples of the application of MAE for the extraction of anthocyanins from different natural matrices. Optimal extraction conditions to achieve maximum yield.

Natural Matrices	Tª (°C)	Solvent (%)	Time (s)	L/S Ratio (mL/g)	Irradiation Power (W)/Solid Amount (g)	**Steps after Separation.** **Comments on the Extract.**	Recovery	Ref.
**Fig *(Ficus carica* L.) peel.**	62.4	Ethanol 100% (pH 3)	300	100:5	400/0.5	Centrifugation, filtration, and evaporation. Purity of the extract: 7.43 mg of C3R/g of extracted residue. It was lower than the obtained by UAE.	411 mg of C3R/100 g of fig peel DW.	[38]
**Lavender *(Lavandula pedunculata* L.) fresh plants.**	-	Water	114.3	30.32:1	464.9/1	Cooling, centrifugation, filtration, and evaporation. No selectivity studies.	273.3 mg of C3G/L.	[55]
**Cranberry.**	50	Ethanol 52% (pH = 3)	8	28:1	-/2	Centrifugation and filtration. No selectivity studies.	306 mg of C3G/100 g of cranberry.	[57]
**Red cabbage.**	100	Water (pH = 3–3.3)	300	30:1	200/5	Cooling and filtration. Extraction efficiency comparable to that of the EC. ∙	110.0 mg of C3G/L.	[58]
**Red cabbage.**	90	Ethanol 50% (pH = 3–3.3)	600	20:1	600/5	Cooling and filtration. Extraction efficiency lower than with EC.	220.2 mg of C3G/L.	[58]
**Purple sweet potato.**	-	Ethanol 30% (citric acid pH = 2)	500	3:1	320/10	Filtration and centrifugation. Anthocyanin composition comparable to that of the extract obtained with aqueous solvent containing citric acid.	31 mg of C3GE/100 g of potato.	[59]
**Black raspberry Korean.**	-	Ethanol 74% (pH = 2)	66	30:1	148/5	Filtration. No selectivity studies.	372 mg of C3G/100 g of fruit.	[60]
**Eggplant Peel.**	-	Ethanol 80%	40	50:1	480/-	Centrifugation and filtration. No selectivity studies.	881 mg of C3G/100 g of peel.	[61]
**Grape** **juice waste.**	55	Double distilled water	138.6	19.2:1	435/1	Cooling to room temperature and filtration. No selectivity studies. ∙	132 mg of M3G/100 g of grape juice waste DW.	[28]
**Blackcurrant.**	-	Ethanol 60% (pH = 2.5)	984	28.3:1	551/-	Cooling and centrifugation. No selectivity studies.	47.37 mg of C3G + D3R + C3R + D3DG/100 g of blackcurrant.	[62]
**Red rice.**	-	Ethanol 85% acidified	100	22:1	400/-	Cooling and centrifugation. No selectivity studies.	3.82 mg of C3G/100 g of rice.	[23]
***Rosa pimpinellifolia* L. fruits.**	60	Ethanol 26.85% (NH₄)₂SO₄ 19.15%	1037.4	40:1	400/20	Cooling and centrifugation. The purity of the extract obtained by MA-ATPE was 1.65-fold greater than MAE using 80% ethanol.	1373.04 mg C3GE/g of fruit DW.	[63]

Note: C3G, cyanidin-3-glycoside; C3GE, cyanidin-3-glycoside equivalent; C3R, cyanidin 3-rutinoside; D3DG, delphinidin 3-O-β-d-glucoside; D3R, delphinidin 3-O-rutinoside; DW, dried weight; MA-ATPE, microwave-assisted aqueous two-phase extraction; M3G, malvidin-3-glucoside. Ref. reference; Tª, temperature.

**Table 3:** Current examples of the application of SFE with CO_2_ for the extraction of anthocyanins from different natural matrices. Optimal extraction conditions to achieve maximum yield.

Natural Matrices	Tª (°C)	P. (bar)	Co-Solvent	Flow Rate	**Steps after Separation.** **Comments on the Extract.**	Recovery	Ref.
Haskap (*Lonicera caerulea* L.) berry pulp paste.	65	450	Water L/S ratio 5.4/3.2 (*w*/*w*)	15 min static time 20 min dynamic time at 10 mL/min.	The extract was collected in a vial free of CO_2_. 527 mg of C3G in the extract by g of C3G in starting material.	25 mg of C3G/g of paste DW.	[68]
Indian blackberry *(Syzygium* *Cumini*) fruit pulp.	50	162	Ethanol (10 g of sample)	2 g/min.	The extract was separated and collected at an ambient temperature and atmospheric pressure. A total of 7 different anthocyanins were extracted together with 8 different bioactive phenols.	231.28 mg C3G/100 g of fruit.	[69]
Colombian blueberry *(Vaccinium meridionale*) fresh and mature fruit.	40	300	None (160 g of sample and 800 g of sample)	32 g/min.	The extract was separated and collected at ambient temperature and atmospheric pressure. No electivity studies. Better yields were obtained from freeze-dried samples using water and ethanol as co-solvent.	26.7 mg of extract/g of sample.	[72]
Bilberry *(Vaccinium myrtillus* L.) dried fruits.	45	250	(1) 6% of 30% distilled water, 70% ethanol. (2) 6% of 50% distilled water, 50% ethanol at 6 mL/min. (3) 9% of 90% distilled water, 10% ethanol. (430 g of sample)	Multistage supercritical/subcritical extraction: (1) SC-CO_2_ 8 kg/h (2) SubC-CO_2_ 6 kg/h (3) SubC-CO_2_ 6 kg/h.	The extract was separated and collected at ambient temperature and atmospheric pressure by a cyclonic separator. SubC-CO_2_ selectively extracted C3G and C3A.	60 mg of C3G/100 g of fruit DW.	[73]
Roselle (*Hibiscus Sabdariffa* L.) dry calyces.	70	89	Ethanol 75% (1.5 g of sample)	6 mL/min (modifier flow rates 9.5%)	Dried at 40 °C to maintain the compounds structure. No selectivity studies. Study the percentage of the red color extracted from Roselle calyces.	26.7 g of dried extract/100 g of sample.	[74]
Merlot red grape (*Vitis vinifera)* pomace.	95	100	Ethanol 10 mL/min (30 g of sample)	32 g/min	The extracts were collected by a cyclone separator system. The extraction efficiency was around 36%. This can increase to 63% if the extraction time is increased from 80 to 180 min.	700 mg of M3G/kg of grape DW.	[75]

Note: C3A, cyanidin-3-O-arabinoside; C3G, cyanidin-3-glucoside; DW, Dried weight; M3G, Malvidin-3-O-glucoside; P., pressure; Ref. reference; SC-CO_2_, supercritical CO_2_ extraction; SubC-CO_2_, subcritical CO_2_ extraction; Tª, temperature.

**Table 4:** Current examples of the application of SWE and PLE for the extraction of anthocyanins from different natural matrices. Optimal extraction conditions to achieve maximum yield.

Natural Matrices	Tª (°C)	P. (bar)	Time (min)	Solvent/Flow Rate (mL/min) (Amount of Sample)	**Steps after Separation.** **Comments on the Extract.**	Recovery	Ref.
**SWE**							
**Raspberry.**	130	70	90	Double distilled water/3 (20 g FW).	The extract was collected and analyzed immediately. The content of individual anthocyanins, C3S, C3G, and C3CS, in the extract was higher for SWE than for hot water extraction and methanol extraction.	815 mg of C3GE/100 g of Raspberry FW.	[79]
**Blueberries.**	130	100	3	Water 1% citric acid)/- (1 g DW).	The extract was filtered using nitrogen gas. The amount of anthocyanins in the extract obtained by SWE was about 4.5 times higher than that obtained by pressed juice and 1.5 times higher than that obtained by hot water.	50 mg of anthocyanin pigment/100 g of blueberries FW. 18 mg of M3G/100 g of blueberries FW.	[80]
**Chokeberries.**	190	100	1	Water 1% citric acid)/- (1 g DW).	The extract was filtered using nitrogen gas. The amount of anthocyanins in the extract obtained by SWE was about 9.5 times higher than that obtained by pressed juice and 1.7 times higher than that obtained by hot water.	66 mg of anthocyanin pigment/100 g of chokeberries FW. 134 mg of C3Ga/100 g of chokeberries FW.	[80]
**Barberry *(Berberis vulgaris)* Fruit.**	157.5	29.64	170	Water.	The extract was filtrated under vacuum condition. No selectivity studies.	9.84 mg C3G/mL of sample.	[81]
**PLE**							
**Purple sweet potatoes.**	90	-	15 (2 cycles)	Ethanol 80% (acidified 0.1% HCl)/- (10 g).	The extract was collected and adjusted the volume by evaporation. In the extract anthocyanin yield followed the order PLE > UAE > CE, which was opposite to the total phenolic and flavonoid yield CE > UAE > ASE. The extract obtained by PLE contained more diacyl anthocyanins and less nonacyl and monoacyl anthocyanins than CE or UEA extracts.	252.34 mg of C3GE/100 g potatoes DW.	[22]
**Jambolan *(Syzygium cumini* L.) fruit.**	90	117.2	5 rinsing time; 10 extraction time/cycle (2 cycles)	Ethanol 80% (acidified 0.1% TFA)/- (4 g).	The extract was concentrated in rotavapor. The extract obtained by PLE presented the lowest proportional abundance of diglucosides of delphinidin, petunidin, and malvidin compared to CE and UAE.	47.05 mg C3GE/g of fruit DW.	[42]
**Broken black bean *(Phaseolus vulgaris* L.) hulls.**	60	100	26	Ethanol: citric acid 30:70 (pH = 3.4)/5 (5 g DW).	The extract was lyophilized. The extract obtained by PLE has the highest value of total monomeric anthocyanins compared to UAE and maceration extraction.	3.96 mg C3GE/g of sample DW.	[82]

Note: C3CS, cyanidin-3-(6′-citryl)–sophoroside; C3G, cyanidin-3-O-glucoside; C3Ga, cyanidin-3-galactoside; C3GE, cyanidin-3- glucoside equivalent; C3S, cyanidin 3-sophoroside; DW, dried weight; FW, fresh weight; M3G, malvidin-3-galactoside; P., pressure; PLE, pressurized liquid extraction; Ref., reference; SWE, sub-critical water extraction; Tª, temperature; TFA trifluoracetic acid.

**Table 5:** Some current examples of the application of HHPE for the extraction of anthocyanins from different natural matrices. Optimal extraction conditions to achieve maximum yield.

Natural Matrices	Tª (°C)	P. (Mpa)	Time (min)	Solvent (Amount of Sample)	Solid/Liquid Ratio	**Steps after Separation.** **Comments on the Extract.**	Recovery	Ref.
**Pansies *(Viola x wittrockiana)*.**	Room Tª	384	15	Ethanol 35% (0.8 g DW)	1:30	The extract was filtered. Anthocyanins are selectively extracted, depending on pressure intensity. The extraction of anthocyanin monoglucosides were optimized at pressures of 200 MPa and the acylglucosides were optimized at 600 MPa.	6.09 mg of C3G/g of flower DW	[86]
**Blueberries *(O’Neal variety)*.**	20	500	15	Acetone/water/acetic acid 70:29.5:0.5 (2g)	-	The extract was centrifuged, filtrated, and evaporated. The extract obtained by HHPE has the highest yields of bioactive compounds and the strongest antioxidant capacity compared to the CE extract.	117.1 mg C3GE/100 g of blueberry extract.	[87]
**Haskap *(Lonicera caerulea)* berry.**	18–22	200	10	Ethanol 60% (acidified HCl0.1%) (1 g)	1:20	The extract was centrifuged. High-pressure affected the monomer composition and anthocyanin content in the extract. C3S5G was not detected at 200 MPa/5 and 10 min. P3DG was not detected at 200 MPa/10 min and 500 MPa/15 min. C3HE was not detected at 400 MPa/20 min.	336 mg C3G/100 g of sample	[88]

Note: C3G, cyanidin-3-glucoside; C3GE, cyanidin-3-glucoside equivalent; C3HE, cyanidin-3-hexoside-ethyl-catechin; C3S5G, cyanidin-3-sophoroside-5-glucoside; DW, dried weight; P., pressure; Ref., reference; Tª, temperature.

**Table 6:** Current examples of the application of PEF for the extraction of anthocyanins from different natural matrices. Optimal extraction conditions to achieve maximum yield.

Natural Matrix	Pulses/Pulses Width/Frequency (Hz)	Electric Field Intensity (kV/cm)	Tª (°C)	**Steps after Separation.** **Comments on the Extract.**	Recovery	Ref.
**By-products of Blueberry.**	10/2 μs/-	20	Room Tª	Centrifugation and the supernatant were analyzed. No selectivity studies. The PEF extract has 0.9% more anthocyanins than the untreated extract.	223 mg of C3GE/L of sample.	[27]
**Blueberry pomace.**	100/2 μs/-	20	Room Tª	Centrifugation and the supernatant were analyzed. D3G, D3A, Pt3G, C3A, P3G, P3A, and M3G were identified. The PEF extract has 61% and 84% more anthocyanins than the HVED and UAE extracts, respectively.	175 mg of/100 g of sample DW.	[95]
**Grape peels.**	25/6 μs/10	25	25	Centrifugation and the supernatant were analyzed. The PEF extract was 34% and 420% richer in anthocyanins than the UAE at 50 °C and water extraction at 70 °C of the extracts, respectively.	78 mg of C3G/mL of sample.	[96]
**Blackcurrant.**	315/100 ms/-	1.32	22	Centrifugation and the supernatant were analyzed. Under the best operating conditions, total monomeric anthocyanins increased by 6% in the extract.	1.38 mg of C3G/g of the extract.	[97]
**Frozen/thawed European blueberry (*Vaccinium myrtillus* L.).**	-/20 µs/20	1	20–25	After the treatment, the sample was pressed, and the juice was obtained as extract. The PEF extract has 8.3% more anthocyanins than the untreated extract.	1750 mg of C3G/L of juice.	[98]
**Pinot Noir (PN) and Merlot (M) grapes.**	-/300 s/344	8	Room Tª	After the PEF treatment, no further treatment was conducted. The PEF treatment applied to the PN sample increased the amount of anthocyanins in the extract by 46%. PEF in the M sample decreased the amount of anthocyanin in the extract by 2.7%, despite prior centrifugation.	81.5 mg of M3GE/L of pinot noir wine or must. 76.92 mg of M3GE/of merlot wine or must.	[101]
**Grapefruit juice.**	-/600 μs/1000	20	40	After PEF treatment, the juice was sonicated. The PEF treatment increased the amount of anthocyanins in the extract by 15%. The PEF+UAE treatment increased the amount of anthocyanins in the extract by 23%.	1.58 mg of C3GE/L of juice obtained after PEF treatment. 1.68 mg of C3GE/L of juice obtained after PEF treatment followed by UAE.	[102]
**Strawberry juice (SJ).**	13/2 μs/155	35	22–46	After the treatment, the juice was cooled at 4 °C. The PEF treatment increased the amount of anthocyanins in the extract by 16.9% compared to untreated extract. This increment was 7.8% and 1.7 compared to the extract obtained by UAE and HPE, respectively.	179.21 mg of Pl3G/L of juice.	[103]
**Spinach juice.**	4/80 μs/1000	9	30	After the treatment, the sample was passed through a sterilized double layer muslin cloth. The PEF treatment increased the amount of anthocyanins in the extract by 8.2% compared to the untreated extract. This increase was increased to 18% when the sample was subjected to a UAE before the PEF treatment.	38.12 mg of M3G/L of juice. 41.31 mg of M3G/L of juice when UAE is applied before PEF treatment.	[104]
**Valuable compounds from blackberries.**	100/10 μs/-	13.3	20–35	Supplementary extraction with hot water at 20 °C (W20) and 50 °C (W50) and with 30% ethanol at 20 °C (EE) was performed in a closed diffusion cell in the dark. The amount of anthocyanins obtained by PEF combined with W50 increases by 53% and 185% compared to the extract obtained by HVED+W50 and UAE+W50, respectively.	100 mg of C3G/100 g of sample when W50 was applied. 90 mg of C3G/100 g of sample when EE was applied. 40 mg of C3G/100 g of sample when W20 was applied.	[105]
**Date palm fruit.**	30/30 μs/10	3	Room Tª	No information. The extract obtained by PEF had 177% more anthocyanins than the extract of untreated sample.	2.08 mg of C3GE/L of sample.	[106]
**Red cherry samples.**	-/20 μs/100	2.5	20	No information. The extract obtained after PEF treatment had 20% more C3G than the extract obtained without treatment.	0.23 mg of C3G/100 g of sample FW. 0.20 mg of C3R/100 g of sample FW. 0.02 mg of Pl3G/100 g of sample FW. 0.10 mg of P3G/100 g of sample FW.	[107]
**Red grapes (Pinot Noir (PN) and Merlot (M).**	-/150 s/178	7	20	After PEF treatment, the must was seeded with selected yeasts (Lallemand); after 11 days of fermentation, the yeast was separated by open decanting. Finally, the wine was analyzed. Relative difference to the untreated sample was 186% for the PN sample and 138% for the M sample.	Maximum absorbance = 0.67 u.a. for PN wine. Maximum absorbance = 1.45 u.a. for M wine.	[108]
**Fresh blueberries.**	24,000/1 μs/-	2	26	After the PEF treatment, samples were blotted with paper towels to remove excess water, were weighed, and subject to anthocyanin extraction by CE with ethanol and further centrifugation. The content of anthocyanins in the blueberries treated by PEF was 10% higher than the content in blueberries in sanitizing solutions.	110 mg of C3GE/g of sample FW.	[109]
**Merlot grapes (*Vitis vinifera*)**	1033/20 μs/50	1.4	Room Tª	Extraction by CE with acidified methanol followed by centrifugation. Four types of extract were obtained: juice without treatment (J); treated juice (PEFJ); juice after 48 h of in contact with untreated berries (J48); and juice after 48 h in contact with treated berries (PEFJ48). M3G, D3G, Pt3G, C3G, MAG, DAG, PtAG, MCG, and PtCG were determined in the juices. In J, anthocyanins were not detected, and in PEFJ, only malvidin derivatives and C3G were detected. The J48 and PEFJ48 contained higher amounts of malvidin derivates than PEFJ, with the amount in PEFJ48 340% higher than in J48.	2.07 mg of M3G+MAG+MCG/100 mL of J48. 0.87 mg of M3G+MAG+MCG+CEG/100 mL of PEFJ. 9.88 mg of M3G+D3G+Pt3G+C3G+MA+DAG+PtAG+MCG+PtCG/100 mL of PEFJ48.	[110]

Note: C3A, cyanidin-3-arabinoside; C3GE, cyanidin-3-glucoside equivalent; C3R, cyanidin-3-rutinoside; D3A, delphinidin-3-arabinoside; DAG, delphinidin-acetyl-glucoside; D3G, delphinidin 3-glucoside; MAG, malvidin-acetyl-glucoside; MCG, malvidin-p-coumaroyl-glucoside; M3G, malvidin 3-glucoside; M3GE, malvidin-3-glucoside equivalents; P3A, peonidin 3-arabinoside; P3G, peonidin 3-glucoside; Pl3G, pelargonidin-3-glucoside; PtAG, petunidin-acetyl-glucoside; PtCG, petunidin-p-coumaroyl-glucoside; Pt3G, petunidin 3-glucoside; pulses, number of pulses applied; pulse widths, duration of each pulse; Ref., reference; Tª, temperature.

**Table 7:** Current examples of the application of EAE for the extraction of anthocyanins from different natural matrices. Optimal extraction conditions to achieve maximum yield.

Natural Matrix	Enzymatic Mixture	pH/Tª (°C)	L:S Ratio/Enzymes:Mixture Ratio/Hydrolysis Time (min).	**Steps after Separation.** **Comments on the Extract.**	Recovery	Ref.
**Saffron tepals**	Pectinex (containing cellulase, hemicellulase, and pectinase).	3.5/4 5	10:1/5:100/120	Centrifugation and supernatant was analyzed. Pectinex extracted 50% more anthocyanins than Cellubrix. The EAE with water extracted 33% more anthocyanins than CE with acidified ethanol.	675 mg of C3G/100 g of saffron tepals extracted.	[119]
**Saffron (*Crocus sativus* L.) tepals.**	Cellulolytic preparation Celluclast BG and hemicellulolytic preparation Xylanase AN (1:1).	4/50	10:1/10:100/145-185	Cooling and centrifugation and filtration of the supernatant. No selectivity study	2.0 g of C3GE/kg of saffron tepals DW.	[121]
**Skin of the Băbească neagră grapes.**	Zymorouge pectolytic enzyme EG from *Aspergillus niger.*	5.0/40	28:1/2:100/60	Centrifugation. UAE with 96% ethanol acidified at 50 °C extracted 68% more anthocyanins than EAE.	2.54 mg of C3G/g of sample DW.	[122]
**Mulberry wine residue.**	Pectinase.	5.9/45	20:1/-/58	Filtration. No selectivity study.	6.04 mg of C3G/g of sample.	[123]
**Leaf of monguba.**	α-Amylase and protease.	6.0/50	10:1/-/160	Centrifugation and filtration. • No selectivity study.	30.59 mg of TA/100 g of sample DW.	[124]
**Seeds (*Adenanthera pavonina* L.)**	Protease and cellulose.	7.0/50	45:5/-/160	Cooling and freeze-drying. EAE improved the recovery of TFC than increased 47%.	14.71 μg of total phenols/g of sample DW.	[125]
**Blueberry.**	Pectinase.	4.5/45	8:1/-/60	Centrifugation. No selectivity study.	2.346 mg of TA/mL of extract.	[126]
**Raspberry (*Rubus idaeus* L.) pomace.**	Ultrazym AFP-L.	-/45	100:15/1:100/60	Cooling and centrifugation; the pomace was extracted by CE and fractionated by SFE. 17% more anthocyanins were quantified in pomace without enzymatic treatment. The Ultrazym EAE increased the C3G content of the extract by 13%.	0.32 mg of C3S+C3G+C3R/g of sample FW.	[127]
**Roselle samples.**	Cellulase solution with exo- and endo-β-1,4-D-glucanases.	4.8/40	40:1/16:100/60	EAE+UAE followed by centrifugation. EAE+UAE increased the anthocyanin content by 1.2% compared to the untreated sample.	676.03 mg of C3G/100 g of sample DW.	[128]
**Raspberry wine residues.**	Pectinase	3/40	30:1/0.16:100/30	UAE+EAE followed by centrifugation, evaporation, and freeze-drying. UAE+EAE obtained 49% and 77% more anthocyanins than EC with acidified ethanol and hot water, respectively.	0.853 mg of C3G/g of sample.	[129]
**Raspberry wine residues.**	Pectic enzyme	-/52	100:1/0.2:100/66	Centrifugation, evaporation, and freeze drying. UAE+EAE obtained 32% and 73% more anthocyanins than EC with acidified ethanol and hot water, respectively.	0.75 mg of C3G/g of sample.	[129]

Note: C3G, cyanidin-3-glucoside; C3GE, cyaniding 3-glucoside equivalents; C3S, cyanidin-3-O-sophoroside; C3R, cyanidin-3-O-rutinoside; DW, dried weight; FW, fresh weight; Ref., Reference; SFE, solid phase extraction; Tª, temperature; TA, total anthocyanins; TFC, total flavonoid content.

**Table 8:** Strengths, weaknesses, and suitability to extract anthocyanins of non-conventional extraction techniques.

Technique	Strengths	Weaknesses	Suitability
**UAE**	Versatile, flexible, low cost, and very easy to use; fast energy transfers; low solvent usage; extraction time (5–60 min); can be combined with heating to improve the yield or with enzymatic treatment to improve the anthocyanin yield and the bioactivity of the extract; available on a large scale.	Lack of homogeneity in the process improved by probe system (PUE); the large-scale application could be limited by the higher cost and nonlinearity of process; after the extraction, a filtration and clean-up step is required; the process can lead to operator fatigue.	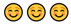
**MAE**	Quick and homogeneous heating; low solvent usage; extraction time (1–40 min); currently, vacuum microwave extraction has been developed to provide a MAE method with a lower reactor temperature; possible application on a large scale.	The solvent must absorb microwaves; the heating could damage the structure and the activity of some compounds; after the extraction, a filtration and clean-up step is required.	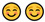
**SFE**	CO_2_ as a solvent; easy to remove after extraction; reduced the thermal degradation. Extraction time (up to 1 h); it does not require an alternative energy source; it is available on a large scale.	Needs a co-solvent to extract polar compounds. The amount and type of co-solvent need to be optimize together with other parameters. SWE present the limitation of need high temperature to reach the subcritical condition, ethanol could be used instead of water.	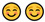
**PLE**	Low solvent consumption; protection for oxygen and light sensitive compounds; it needs temperature; possible application on a large scale.	Expensive equipment required; after the extraction, a clean-up step is required; extraction time (1–2 h).	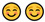
**HHPE**	Short extraction time (~ 5 min); performed at room temperature; higher repeatability; smaller amount of solvents; possible application at large scale.	High investment cost and cost maintenance and service; high pressure could affect the structure or activity of some compounds. The parameter should be optimized to avoid it.	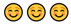
**PEFE**	Short extraction time (less than 1 s); performed at room temperature; low energy and monetary costs; possible application on a large scale.	Some compounds could be affected by high electric fields; it is desirable to reduce the electrical conductivity of the matrix before the extraction. For industrial application there are some problems related to: non-uniform distribution of the electric pulses, the suitable solvents are very limited and cooling system is necessary to control the temperature when extracting thermolabile compounds if high electrical pulses are applied.	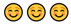
**HVED**	Low temperature; short extraction time and energy input; possible application on a large scale.	High cost maintenance and service; high voltage electrical discharges may generate chemical products and free reactive radicals, which can react with antioxidant compounds decreasing their bioactive activity.	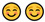
**EAE**	Moderate extraction conditions; eco-friendly; selectivity due to the specificity of enzymes; can be combined with ultrasonic extraction to improve the yield and the bioactivity of the extract.	Expensive cost of enzymes; activity of enzymes varying with the pH, temperature and nutrients of the matrix; after the extraction, a filtration and clean-up step is required. Difficulties to be applied on a large scale; extraction time (1–12 h); low availability of commercial enzyme types; sometimes they have low selectivity and variability.	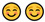

## Data Availability

The data presented in this study are available in review.
